# Molecular evidence for the bi-clonal origin of neuroendocrine tumor derived metastases

**DOI:** 10.1186/1471-2164-13-594

**Published:** 2012-11-05

**Authors:** Beate Rinner, Birgit Gallè, Slave Trajanoski, Carina Fischer, Martina Hatz, Theresa Maierhofer, Gabriele Michelitsch, Farid Moinfar, Ingeborg Stelzer, Roswitha Pfragner, Christian Guelly

**Affiliations:** 1 Center for Medical Research, Medical University of Graz, Stiftingtalstraße 24, Graz, 8010, Austria; 2 Institute of Pathology; Medical University of Graz, Auenbruggerplatz 26, Graz, 8036, Austria; 3 Clinical Institute of Medical and Chemical Laboratory Diagnostics; Medical University of Graz, Auenbruggerplatz 15, Graz, 8036, Austria; 4 Institute of Pathophysiology and Immunology; Medical University of Graz, Heinrichstraße 31a, Graz, 8010, Austria

**Keywords:** Neuroendocrine tumors, Clonality of metastases, Somatic mutations

## Abstract

**Background:**

Reports on common mutations in neuroendocrine tumors (NET) are rare and clonality of NET metastases has not been investigated in this tumor entity yet. We selected one NET and the corresponding lymph node and liver metastases as well as the derivative cell lines to screen for somatic mutations in the primary NET and to track the fate of genetic changes during metastasis and *in vitro* progression.

**Results:**

Applying microarray based sequence capture resequencing including 4,935 Exons from of 203 cancer-associated genes and high-resolution copy number and genotype analysis identified multiple somatic mutations in the primary NET, affecting *BRCA2, CTNNB1, ERCC5, HNF1A, KIT, MLL, RB1, ROS1, SMAD4,* and *TP53*. All mutations were confirmed in the patients’ lymph node and liver metastasis tissue as well as early cell line passages. In contrast to the tumor derived cell line, higher passages of the metastases derived cell lines lacked somatic mutations and chromosomal alterations, while expression of the classical NET marker serotonin was maintained.

**Conclusion:**

Our study reveals that both metastases have evolved from the same pair of genetically differing NET cell clones. In both metastases, the *in vivo* dominating “mutant” tumor cell clone has undergone negative selection *in vitro* being replaced by the “non-mutant” tumor cell population. This is the first report of a bi-clonal origin of NET derived metastases, indicating selective advantage of interclonal cooperation during metastasis. In addition, this study underscores the importance to monitor cell line integrity using high-resolution genome analysis tools.

## Background

Tumour metastasis is a multistage process in which malignant cells spread from the initial tumour to colonize the same or distant organs [1]. Sequential events during metastasis involve local invasion, intravasation, and survival in the circularization, extravasation and colonization of the host organ. An important variable is the temporal course of metastasis. Whereas breast cancer recurrences are often detected following decades of remission, lung cancers establish distant metastases within months of diagnosis [2-4]. Apparently, the capacity of cancer cells to infiltrate distinct organs and to develop macrometastases does not coincide. It is hypothesized, that during this period of metastatic latency disseminated cancer cells acquire the ability to colonize the host organ [1]. Among other factors like immunosurveillance, switches of transcriptional pathways and programmes, the local microenvironment is considered a critical determinant of metastatic outgrowth. During the latency period a local malignant (co)evolution of the disseminated cancer cell and/or the seeded microenvironment might be required for effective colonization and metastatic outgrowth. To date dissemination and metastatic outgrowth has mainly been attributed to clonally derived cancer cells. Even in advanced genetically heterogeneous primary tumors metastases appear to be of clonal origin [5,6], although concepts of interclonal cooperativity have been considered and tested in model systems [7-9].

In a recent report, [10] massive parallel DNA sequence analysis revealed statistical evidence for the multiclonal origin of breast cancer metastases. By comparison of the mutation spectrum of a breast cancer patient’s primary and secondary tumors, the prevalence of specific mutations was found significantly decreased or increased relative to the primary tumor. It was suggested that the patient’s metastases have been formed by at least three different cell clones.

We have chosen three recently described [11] novel cell lines established from a metastatic midgut (terminal ileum) neuroendocrine tumor (NET) of the same patient together with the corresponding original tissue samples: 1) P-STS, a cell line established from the primary tumor (P-Tu), 2) L-STS, from a lymph node metastasis, and 3) H-STS, a cell line established from a liver metastasis, to screen for mutations in the P-Tu and to explore the evolution of somatic mutations and corresponding cell population kinetics during *in vitro* cultivation.

## Results

### Targeted resequencing

Using the hg18 reference sequence (ucsc genome db) we have designed a custom sequence capture array (NimbleGen 385 k technology) including 4,935 Exons from 203 cancer associated genes based on the information available via the Cancer Gene Census (http://www.sanger.ac.uk/genetics/CGP/Census/) [12] and 16 collagen genes, comprising a total of 1.4 Mb of genomic information for enrichment and subsequent 454 GS FLX Titanium based parallel re-sequencing (Additional file 1: Table S1). Primarily, only cancer-associated genes known for and characterised by missense mutations, stop-mutations, small and large in/del mutations as well as frame-shift mutations were included in the design of the capture array.

**Figure 1:**
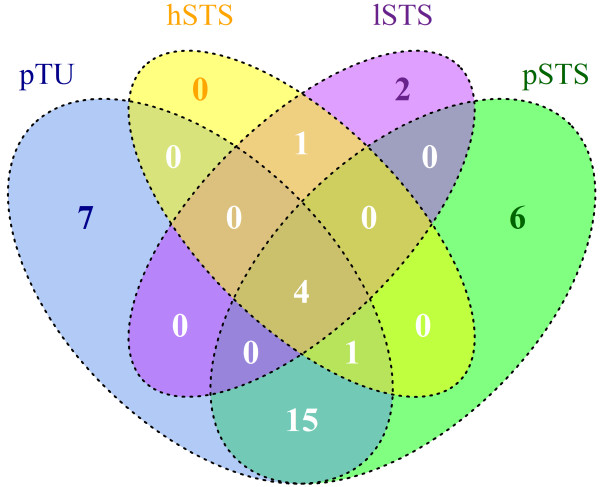
Venn diagram of P-STS, H-STS, L-STS: Four genes (SDHB, COL4A3, LIFR, HIST1H2BA) with a common mutation over all screened samples could be identified.

Sequencing of ~ 70 Mb of target-enriched genomic information per sample (Table 1 A) was sufficient to achieve an average of > 25-fold coverage of the target region. 63-79% of the reads mapped to the target region with > 80% of target bases covered at least 5-fold (Table 1 A). 4,489 high-confidence sequence variants were detected for the P-Tu sample using the standard GS Reference Mapper tool (UCSC hg18 built, SNPdb built130), with 3,961 of these representing known variants. 280 variants were detected within the exonic sequences. 27 of these variants were novel; 24 thereof non-synonymous. (Table 1 B and Additional file 1: Table S2 A). We could identify four genes (SDHB, COL4A3, LIFR, HIST1H2BA) with known mutations observed in all samples as can be seen in the Venn diagram (Figure 1). The fraction of non-synonymous to synonymous sequence variants among the newly identified variants was disproportionately higher (> 5-fold) compared to the ratio within the known variants (Table 1 B) indicating a substantial fraction of these variants to be of somatic origin. Although all samples were sequenced to approximately the same coverage, the L-STS p49 and H-STS p42 cell lines lacked the variants identified in the P-Tu and did not show sample specific variants (Table 1 B and Additional file 1: Table S2 c and d). A high level of sequence capture efficiency and reproducibility among the processed samples is shown for a representative locus (*BRCA2;* Additional file 1: Figure S1).

**Table 1:** Statistics on sequence capture based resequencing

**A)**							
	**p-TU**	**P-STS -P41**	**H-STS -P42**	**L-STS -P49**			
Total number of reads	272,728	270,044	256,619	268,969			
Numb. of mapped reads	262,773 (96.35%)	259,781 (96.20%)	247,300 (96.37%)	258,908 (96.26%)			
NumUniqueInRegions (reads within target region	184,206 (72.08%)	200,711 (78.60%)	192,918 (79.38%)	160,272 (63.75%)			
Average coverage	~29.3-fold	~31.9-fold	~30.7-FOLD	~25.5-FOLD			
% target bases covered ≥ 5-fold	>89%	>86%	>81%	>82%			
**B)**							
	**p-TU**	**P-STS -P41**	**H-STS -P42**	**L-STS -P49**			
HC Diffs (total)	4489	3756	3541	4631			
HC Diffs (known)	3961	3372	3163	3907			
HC DIffs (within cds)	280	251	206	213			
novel or pathogenic (in coding seq.)	27	26	6	7			
non-synonymous	24	19	5	6			
synonymous	3	7	1	1			
knowing (in coding seq.)	253	225	200	206			
non-synonymous	97	80	77	77			
synonymous	156	145	123	129			
**C)**							
**GeneID**	**Status**	**Mut. type**	**Nucleotide**	**AA**	**Refseq ID**	**Chr.**	**Position**
BRCA2	unknown	missense	c.C1783T	H595Y	NM_000059.3	13q	31805398
ERCC5	unknown	missense	c.T266C	V89A	NM_000123.2	13q	102304109
KIT	unknown	missense	c.A1606C	M536L	NM_000222.1	4q	55288206
MLL	unknown	missense	c.C11305T	H3769V	NM_005933.2	11q	117895710
ROS1	unknown	missense	c.G625A	D209N	NM_002944.2	6q	117824925
SMAD4	unknown	missense	c.G290A	R97H	NM_005359.5	18q	46829094
SDHB	unknown	missense	cC98T	A33L	Nm_003000.2	1p	17243945
HNF1A	unknown	frameshift	c.872_873insC	p.G292fs*25	NM_000545.4	12q	119916507
RB1	known	missense	c.211_213insAG	p.A74fs*3	NM_000321.2	13q	47779500
TP53	known	missense	c.C817T	R273C	NM_000546.4	17q	7517846
TP53	rs1042522	missense	c.C215G	R72P	NM_000546.4	17q	7520197
HNF1A	rs56348580	synon.	cG864C	G288G	NM_000545.4	12q	119916500
KIAA1549	rs59985563	missense	cA1090G	T364A	NM_001164665.1	7q	138253822
KIAA1549	rs61734132	missense	cC1940G	S647C	NM_00164665.1	7q	138252972

Sequencing statistics (A) and target region related detection of High Confidence Differences (B). C: Selected sequence variants (known mutations, unknown variants and SNPs) further pursued by complementary methods. Chromosomal positions according to Ensembl database release 54 from May 2009.

### Sanger sequencing of BRCA2, CTNNB1, ERCC5, HNF1A, KIT, MLL, RB1, ROS1, and SMAD4

Six of the novel heterozygous missense variants (*BRCA2, ERCC5, KIT, MLL, SMAD4,* and *ROS1)* and three frameshift sequence variants of *RB1*, *HNF1A* and *CTNNB1* identified in the P-Tu sample were selected for further investigation (Table 1 C). None of these mutations was detectable in blood derived DNA of the same patient by Sanger sequencing proving the somatic origin of the mutations. All variants were heterozygous and detectable in both metastases (9/9 variants) and the corresponding cell lines H-STS (9/9) and P-STS (9/9) at lower passages (passage 7 for H-STS, and 14 for P-STS; Table 2) whereas the L-STS at passage 9 has already lost the mutant alleles and displayed only reference/wild-type allele information (Table 2) for all nine investigated markers. None of these sequence variants initially detected in the P-Tu tissue and in cell line P-STS was found in the metastasis derived cell lines L-STS and H-STS at higher passages (passages 41, 49, and 42 respectively).

**Table 2:** Difference confirmation by sanger sequencing

somatic variations	missense	MLL:NM_005933	P-TU	het C/T	H-STS	het C/T	L-STS	het C/T
		cC11305T (H3769V)	P-STS P14	het C/T	H-STS P7	het C/T	L-STS P9	hom C (wt)
			P-STS P41	het C/T	H-STS P42	hom C (wt)	L-STS P49	hom C (wt)
		BRCA2: NM_000059	P-TU	het C/T	H-STS	het C/T	L-STS	het C/T
		cC1783T (H595Y)	P-STS P14	het C/T	H-STS P7	het C/T	L-STS P9	hom C (wt)
			P-STS P41	het C/T	H-STS P42	hom C (wt)	L-STS P49	hom C (wt)
		ERCC5: NM_000123	P-TU	het T/C	H-STS	het T/C	L-STS	het C/T
		cT266C (V89A)	P-STS P14	het T/C	H-STS P7	het T/C	L-STS P9	hom T (wt)
			P-STS P41	het T/C	H-STS P42	hom T (wt)	L-STS P49	hom T (wt)
		KIT: NM_000222	P-TU	het A/C	H-STS	het A/C	L-STS	het A/C
		cA1606C (M536L)	P-STS P14	het A/C	H-STS P7	het A/C	L-STS P9	hom A (wt)
			P-STS P41	het A/C	H-STS P42	hom A (wt)	L-STS P49	hom A (wt)
		TP53: NM_000546	P-TU	het C/T	H-STS	het C/T	L-STS	het C/T
		cC817T (R273C)	P-STS P14	het C/T	H-STS P7	het C/T	L-STS P9	hom C (wt)
			P-STS P41	het C/T	H-STS P42	hom C (wt)	L-STS P49	hom C (wt)
		SMAD4: NM_005359	P-TU	het G/A	H-STS	het G/A	L-STS	het G/A
		cG290A (R97H)	P-STS P14	het G/A	H-STS P7	het G/A	L-STS P9	hom G (wt)
			P-STS P41	het G/A	H-STS P42	hom G (wt)	L-STS P49	hom G (wt)
		ROS1: NM_002944	P-TU	het G/A	H-STS	het G/A	L-STS	het G/A
			P-STS P14	het G/A	H-STS P7	het G/A	L-STS P9	hom G (wt)
			P-STS P41	het G/A	H-STS P42	hom G (wt)	L-STS P49	hom G (wt)
	frame shift	HNF1A: NM_00545.4	P-TU	het C ins		het C ins	L-STS	het C ins
			P-STS P14	het C ins		het C ins	L-STS P9	wt
			P-STS P41	het C ins		wt	L-STS P49	wt
		CTBBB1: NM_001098209.1	P-TU	het GTT del	H-STS	het GTT del	L-STS	het GTT del
			P-STS P14	het GTT del	H-STS P7	het GTT del	L-STS P9	wt
			P-STS P41	hom GTT del	H-STS P42	wt	L-STS P49	wt
germline transmited variants	SNP	HNF1A: NM_000545.4	P-TU	hom C	H-STS	hom C	L-STS	hom C
		cG864C (G288G)	P-STS P14	hom C	H-STS P7	hom C	L-STS P9	hom C
		rs 56348580	P-STS P41	hom C	H-STS P42	hom C	L-STS P49	hom C
		TP53: NM_000546	P-TU	het C/G	H-STS	het C/G	L-STS	het C/G
		cC215G (P72R)	P-STS P14	het C/G	H-STS P7	het C/G	L-STS P9	het C/G
			P-STS P41	homG	H-STS P42	het C/G	L-STS P49	het C/G
		KIAA1549: NM_001164665.1	P-TU	homG	H-STS	homG	L-STS	homG
		cA1090G (T364A)	P-STS P14	homG	H-STS P7	homG	L-STS P9	homG
		rs59985563	P-STS P41	homG	H-STS P42	homG	L-STS P49	homG
		KIAA1549: NM_001164665.1	P-TU	homG	H-STS	homG	L-STS	homG
		cC1940G (S647C)	P-STS P14	homG	H-STS P7	homG	L-STS P9	homG
		rs61734132	P-STS P41	homG	H-STS P42	homG	L-STS P49	homG
	missense	SDHB: NM_003000.2	P-TU	het C/T	H-STS	het C/T	L-STS	het C/T
		cC98T (A33L)	P-STS P14	het C/T	H-STS P7	het C/T	L-STS P9	het C/T
			P-STS P41	het C/T	H-STS P42	het C/T	L-STS P49	het C/T
		NOTCH1: NM_017617.3	P-TU	het G/T	H-STS	het G/T	L-STS	het G/T
		cG5457T (E1818D)	P-STS P14	het G/T	H-STS P7	het G/T	L-STS P9	het G/T
			P-STS P41	het G/T	H-STS P42	het G/T	L-STS P49	het G/T
		MYC: NM_002467.4	P-TU	het T/C	H-STS	het T/C	L-STS	het T/C
		cT64C (F22L)	P-STS P14	het T/C	H-STS P7	het T/C	L-STS P9	het T/C
			P-STS P41	het T/C	H-STS P42	het T/C	L-STS P49	het T/C

Sequencing results as determined for the selected missense, frame-shift and SNPs variants in the primary tumor (P-TU), the metastatic tissues (H-STS and L-STS) and early and late passages of the respective tissue-derived cell lines. Het: heterozygous genotype; Hom: homozygous genotype; wt: wild-type e.g. same seq. information as observed for blood DNA.

### High-resolution melting curve analysis

To evaluate, whether the sequence variants might still occur albeit at lower frequency eventually in form of a minor (background) cell population, we implemented high-resolution melting curve analysis for MLL (missense variant) and RB1 (frameshift mutation). Using mutation specific HRM assays with a 10% minor variant detection limit, we confirmed heterozygosity of the variants in the P-Tu sample as well as the higher passages of P-STS sample and the absence of a ≥ 10% subpopulation in the L-STS p49 and H-STS p42 cell lines (Additional file 1: Figure S2).

### Sequencing of *TP53* variants

Two *TP53* sequence variants (SNP rs1042522, p.R72P and mutation c.C817T encoding p.R273C; Table 1 C) were identified in the P-Tu sample, whereas the data from the P-STS p41 indicated homozygous mutant *TP53* sequence information (100% of the reads; total of 28 reads). The *TP53* c.C817T mutant was not detectable in L-STS p49 and H-STS p42 by next-generation sequencing, revealing only wild-type sequence reads. The known *TP53* SNP rs1042522 was confirmed heterozygous in the blood DNA sample, whereas the c.C817T mutation was not detectable in this reference DNA, validating the somatic origin of the known *TP53* mutation. Accordingly, Sanger sequencing confirmed heterozygosity for both *TP53* sequence variants in the P-Tu and the metastatic tissues (liver and lymph node). As observed for the previously described gene panel, H-STS p42 and L-STS p49 cell lines exhibited a loss of the mutant allele information at higher passages and in H-STS p7. In contrast, P-STS p41 exclusively exhibited the *TP53* sequence variant at higher passages, which was a result of a copy neutral loss of heterozygosity after passage 14, as determined by complementary methods (e.g. microarray based CNV analysis; Additional file 1: Table S4). Complete Submission SRA055939; pTU SRR521279; hSTS SRR521655; pSTS SRR521658; lSTS SRR521659.

### Chromosomal copy number (CN) analysis using Affymetrix 6.0 CNV/SNP arrays

To evaluate, whether chromosomal imbalances (especially loss of heterozygosity; LOH) might have contributed to the selective loss of the variant alleles we performed copy number (CN) and genotype analysis using Affymetrix 6.0CNV/SNP Arrays (Additional file 1: Table S4). We did not observe any CN alterations for the L-STS p49 and the H-STS p42 cell lines. The primary NET (P-Tu) displayed a single copy neutral LOH at chr.3: p26.3-p21.1, whereas the P-STS p41 showed gain of chromosome arms 1p and 17q-p11 and loss of 3p26.3-p11.2, 4q35.1-qter, 11q23.2-q23.3 and 20p13-p12.1 (Additional file 1: Figure S3). Both metastases tissues (L-Met and H-Met) displayed the same copy neutral LOH at chr.3 as the P-Tu, without the indication of further chromosomal alterations. CN data are summarized in Additional file 1: Table S4. In-depth analysis of the CN probes and genotypes at the genetic loci of BRCA2, ERCC5, KIT, MLL, SMAD4, ROS1, RB1, TP53 and HNF1A revealed unchanged CN (n = 2) with interspersed heterozygous genotypes (Additional file 1: Figure S4 a-i). In contrast, the expected loss-of-heterozygosity (LOH) at the TP53 locus of the P-STS sample turned out to be a copy number neutral LOH, resulting from an early loss of the wild-type allele followed by re-duplication of the mutant allele (Additional file 1: Figure S4 e) (complete array data are accessible trough GEO Series accession number GEO Accession Nummer: GSE39371; GSM966942 primary tumor p-TU; GSM966943 cell line H-STS; GSM966944 cell line L-STS; GSM966945 cell line P-STS).

### Immunofluorescence analysis for serotonin (5-HT) on a confocal scanning microscope

**Figure 2:**
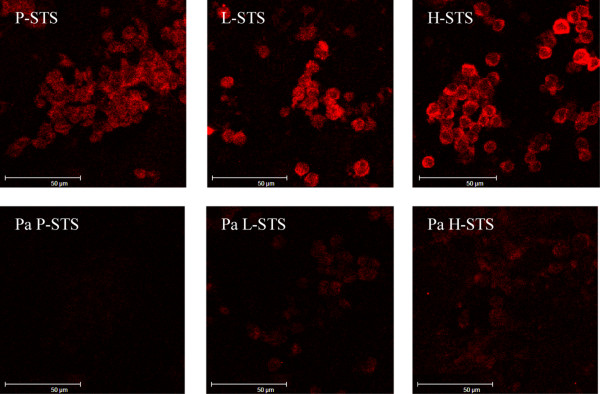
**Immunodetection of serotonin expression at higher cell passages (P-STS P49, L-STS P42 and H-STS P41).** The KRJ neuroendocrine tumor cell line was used as positive control (data not shown). Preadsorption (pa) with serotonin creatinine sulphate complex (pa P-STS, pa L-STS, pa H-STS) was done to obviate unspecific binding.

Immunocytochemical characterization in former studies [11] showed an expression of pancytokeratin, cytokeratins 7, 8, 18 and 19, serotonin (5-HT), NSE, CD56, protein gene product 9.5 (PGP9.5), calcitonin, synaptophysin, and gastrin-releasing factor in the P-STS cell line. The L-STS cell line was characterized for positive immunoreactivity with cytokeratin 8/18 (CK8/18) and PGP 9.5. The H-STS cell line showed a positive immunocytochemical reactivity for chomogranin (CG), serotonin (5-HT), PGP9.5 and CK8/18. The NET cell status at higher cell passages was confirmed by immunodetection of serotonin expression by confocal laser scanning microscopy (Figure 2 upper panel). Furthermore, unspecific binding was excluded by preadsorption with 1 mM serotonin creatinine sulphate complex which resulted in a suppression of the staining (Figure 2 lower panel).

## Discussion

While chromosomal aberrations are frequently found in midgut NETs [13-15], reports on the observation of frequent gene mutations (*SDHD, K-ras*) are rare. By virtue of sequence capture based high-throughput sequencing targeting 203 known cancer associated genes; we have identified multiple *de-novo* somatic mutations in a primary midgut NET (P-Tu). Mutations affect genes involved in the regulation of the cell cycle (*TP53* and *RB1*), apoptosis, senescence and DNA repair (*BRCA2*, *ERCC5* and *TP53*), transcriptional regulation (*MLL*, *SMAD4* and *HNF1A*) and cell signalling (*CTNNB1, KIT* and *ROS1*). Whereas these tumor-specific mutations were conserved in the patient’s lymph node- and liver metastases, the heterozygous sequence variants were lost in metastases derived cell lines (H-STS and L-STS), indicating a lack of selective pressure to maintain the mutations (probability of < 0.1% for the random loss of 10/10 mutations) during *in vitro-*cultivation. Notwithstanding several prominent mutations in the DNA repair genes, no increase in the number of mutations or CNVs was observed in the L-STS and H-STS, whereas the P-STS acquired multiple chromosomal alterations during *in vitro* cultivation not evident in the P-Tu.

The only chromosomal alteration observed for the P-Tu and the metastases is a copy neutral LOH on chromosome 3p21-p26. Among the DNA mismatch repair gene MLH1 (3p21.3-p23) and the DNA repair gene XPC (3p25.23), which is frequently found mutated in Xeroderma pigmentosum syndrome type C [16], this locus contains the VHL (von Hippel-Lindau) tumor suppressor gene. Germline mutations of VHL have been reported in patients with the von Hippel-Lindau disease, a familial cancer syndrome. All three genes were covered by the sequence capture array and sequenced to sufficient depth to identify eventually occurring variants in coding sequences. Nonetheless, no exonic sequence variants were observed for these genes, although any variants affecting gene regulatory regions might be missed by our enrichment approach.

The P-STS cell line acquired several aberrations frequently observed in lung and midgut NETs (loss of chromosome 3p, gain of 17q and mutation of *TP53*) [13]. Reduplication of one parental allele coinciding with loss of the other allele inevitably leads to a copy neutral LOH indicated by homozygosity and a CN of 2 at the respective region. We observed this event at the *TP53* locus (chr.17p11-ter) during passaging of the P-STS cell line. It has been shown in a previous study that tumorigenicity of both metastases derived cell lines (20% and 60% for L-STS p45 and H-STS p40) in SCID mice was substantially decreased compared to the P-STS p25 (100% tumorigenicity) cell line [11]. Poor survival and increased aggressiveness in lung NET patients have been attributed to mutations in the *TP53* gene [17], which might be mirrored by the differing tumorigenicity of the cell lines in SCID mice and the concordant bi-allelic deletion of *TP53* in the P-STS cell line.

Heterozygosity at the *BRCA2, CTNNB1, MLL, SMAD4, ERCC5, RB1* and *KIT* chromosomal loci and a CN of 2 indicate that another mechanism must have lead to the loss of the mutations during *in vitro* cultivation. Maintenance of serotonin expression in the L-STS and the H-STS throughout cell passaging proves a classical NET characteristic [18] of the cell lines. It is highly unlikely that all the DNA alterations observed in the primary tissues were selectively repaired during *in vitro* cell expansion almost in parallel (in early passages) and indepently in both cell lines (L-STS and H-STS). We presume that a minor NET cell population bearing none of the mutant alleles, which must have already been present during metastasis, has overgrown and replaced the initially dominating mutant H-STS and L-STS cell lines in parallel. This hypothesis implies the intriguing fact that metastasis formation and/or outgrowth must have involved a multiclonal complex comprising at least two different NET cell populations. This could have happened in two ways, either by dissemination of a multicellular complex consisting of at least two genotypically different NET subclones or by a stepwise process where the newly established metastasis has attracted other circulating tumor cells to associate. It is likely that the reported different tumorigenic properties in SCID mice are directly linked their genomic alterations. Additionally, the parallel finding of metastatic bi-clonality might indicate commensal biological tasks during metastases formation. Various biological effects might underlie this interdependence. A non-mutant (or less mutant) tumor cell population will be less immunogenic thereby protecting early metastatic outgrowth. Functional studies deploying mixtures of clonally expanded metastases and/or primary tumor derived subpopulations in immunodeficient mice might be suited to further investigate the biological principles in an appropriate environment.

## Conclusion

In our study, we demonstrate the bi-clonal origin of two metastases of a NET patient using DNA-enrichment followed by massive parallel resequencing and based on *in-vitro* selection identify and genetically characterize the clonal partners. In our discussion, we propose a model of clonal interdependence during metastases formation as the underlying biological mechanism. Nevertheless, interclonal dependence during metastasis formation might offer another avenue for the development of anti-cancer strategies.

## Methods

### Cell lines and cultivation

P-STS cells were cultured in Ham´s F12:M199 (PAA Laboratories, Pasching, Austria) (1:1) supplemented with 10% FBS (PAA). KRJ-I [16] were cultured in Ham’s F12 medium. The two metastatic cell lines L-STS and H-STS were cultured in serum free Quantum 263 Complete Medium (PAA). All cells were kept in a 5% CO2 atmosphere at 37°C. An adequate review of the immunocytochemical analysis for NET markers especially in the low passages was done previously (Pfragner et al. 2009). Cell cultures were periodically checked for mycoplasma contamination. During this study, cell line batches from low passage (< 15) and high passages (> 15) were used.

### Confocal laser scanning microscopy

Suspensions of P-STS, L-STS, and H-STS were analyzed for serotonin (5_HT) immunoreactivity. The KRJ-I NET cell line served as positive control (data not shown). Negative controls were incubated in buffer instead of the primary anti-serotonin antibody. The primary anti-serotonin antibody was preadsorbed at a dilution of 1:2.000 in 1 mM serotonin creatinine sulphate complex (Sigma, Vienna, Austria) for 1h at 37°C. For immunofluorescence, cells were fixed in 2% paraformaldehyde for 15 min, incubated in.blocking solution for 30 min at RT and incubated for 16–24 hours at 4°C with the primary antibody (polyclonal rabbit-anti-human serotonin; Acris Immunostar, Hudson, WI). After incubation with goat-anti-rabbit-Cy3 antibody (Jackson, Suffolk, UK) for 2 h at RT in the dark the cells were analysed on a confocal laser scanning microscope (Leica TCS SP2; Leica Lasertechnik GmbH, Heidelberg, Germany). Scanning parameters were adjusted for each cell type using its pre-adsorption control.

### Sequence capture array design

A custom tiling NimbleGen 385 k sequence capture array targeting the exonic sequences (n = 4,935) of 203 cancer associated genes and 16 collagen genes were designed and manufactured by Roche NimbleGen. The array was designed using NimbleGen’s standard 15-mer frequency masking to minimize repeat content within capture probes. The probe spacing, tiling overlap, and probe length were determined by NimbleGen using proprietary algorithms. A GFF- or BED-formatted file (Additional file 2 and 3) allowing visualization of the tiled intervals by the Genome Browser (http://genome.ucsc.edu/).

### Sequence capture library construction

Genomic DNA (20 μg per sample) isolated from the snap-frozen primary tumor sample and the three corresponding cell lines (P-STS, H-STS and L-STS) was processed into a capture library according to the manufacturers protocol (Additional file 1: Procedure S1).

### Capture array handling

Hybridization was performed using microarrays merged with X1 mixer on the NimbleGen Hybridization System for 3 days at 42°C following the manufacturer’s recommended conditions (Additional file 1: Procedures S2). Quantitative PCR (SYBR-Green based; LC480 instrument) using four internal NimbleGen control loci (NSC-0237, NSC-0247, NSC-0268, NSC-0272) was performed to estimate relative fold-enrichment (data not shown).

### GS FLX sequencing

The amplified capture libraries were processed into sequencing libraries for the 454 GS-FLX using the Shotgun DNA Titanium Library Construction Kit and low-molecular-weight DNA (without the nebulisation step) protocols (454 Life Sciences, Branford, CT) according to the manufacturer’s recommended conditions. Each captured sample library was sequenced using a quarter of a Titanium PicoTiterplate (70x75) run on the GS-FLX platform.

### Data analysis - Variant detection and annotation

High Confidence Differences were calculated using the GS Reference Mapper assembly package (version 2.0.00.20; Roche Diagnostics) and hg18 reference sequence and SNPdb built 130 (Additional file 1: Procedures S3).

### Sanger sequencing

Sequence variants observed by NGS sequencing were re-evaluated by Sanger sequencing. PCR products were purified using Nucleo Fast® (Macherey-Nagel, Düren, Gemany) clean up PCR plates according to the manufacturer´s protocol. Following purification, capillary sequencing reactions were performed using Applied Biosystems BigDye Terminator v3.1 Ready Reaction Cycle Sequencing Kit (Foster City, CA). Sequencing reactions were purified using Sigma Spin Post Reaction Clean-up Plates (Sigma-Aldrich, Austria) and run on an Applied Biosystems 3730. Data files were analyzed using ABI SeqScape v2.5 software (Applied Biosystems).

### High resolution melting curve analysis

High Resolution Melting Curve analysis for variant positions in RB1 exon 2 and MLL exon 32 mutations was performed using a LightCycler 480 system (Roche Diagnostics, Penzberg, Germany). Primers were designed using primer3 software to span the positions of interest with product sizes of 134 bp for RB1 and 185 bp for MLL. The 20 μl reaction mix for PCR amplification contained 20 ng genomic template DNA, 10 μl of LightCycler480 High Resolution Master Mix (Roche Diagnostics, Penzberg, Germany), 10 μM of each primer and MgCl2 in a final concentration of 2.5 mM. The reaction condition included a pre-incubation step at 95°C for 10 min for the activation of the polymerase, followed by 45 cycles of 95°C for 10 s, 58°C for 15 s and 72°C for 15 s. A melting pre-hold step was included to ensure that all PCR products have re-associated and encourage heteroduplex formation. The melting interval ranged from 65°C to 95°C and increased at 1°C per second with 35 acquisitions per degree. Normalized, temperature-shifted melting curves carrying sequence variation were analyzed using the automated grouping functionality provided by the LightCycler480 GeneScanning 1.5 Software (Roche Diagnostics, Penzberg, Germany).

### Affymetrix SNP 6.0 array processing and analysis

Affymetrix GeneChip Human Mapping SNP 6.0 arrays were performed as described in the Genome-Wide Human SNP Nsp/Sty 6.0 User Guide (Affymetrix Inc., Santa Clara, CA, USA). Detailed information: Additional file 1: Procedures S4.

SNP 6.0 data were imported and normalized using the Genotyping Console 4.0 program default settings. All samples passing QC criteria were subsequently genotyped using the Birdseed (v2) algorithm. We used 60 raw HapMap data generated with the Affymetrix Genome-Wide Human SNP Array 6.0 as reference. Data were obtained from Affymetrix (Affymetrix, Santa Clara, CA) web site and used for normalization.

## Competing interests

The authors declare that they have no competing interests.

## Authors’ contributions

BR carried out the cell culture and drafted the manuscript. BG carried out the molecular genetic studies and drafted the manuscript. ST performed the statistical analysis. CF, MH, TM and GM carried out the molecular genetic studies and participated in the sequence alignment and have been involved in drafting the manuscript. FM and IS carried out the immunhistochemistry. RP established the cell lines and GC designed and drafted the manuscript. All authors read and approved the final manuscript.

## Supplementary Material

Additional file 1 Molecular evidence for the bi-clonal origin of neuroendocrine tumor. Click here for file

Additional file 2 BED File. Click here for file

Additional file 3 GFF File. Click here for file
