# Supplementary material for: Molecular evidence for the bi-clonal origin of neuroendocrine tumor derived metastases
Source: BMC Genomics. 2012 Nov 5;13:594. doi: 10.1186/1471-2164-13-594 (PMC3500212; doi:10.1186/1471-2164-13-594)
Supplement: Additional file 1 — Molecular evidence for the bi-clonal origin of neuroendocrine tumor. [file 1471-2164-13-594-S1.doc]

**Supplemental Text and Figures**

**Molecular evidence for the bi-clonal origin of neuroendocrine tumor derived metastases**

Rinner et.al.

**Supplemental Table 1:** List of 219 sequence capture target genes.

| **Nr.** | **Associated Gene Name** | **Entrez Gene ID** |
| --- | --- | --- |
| 1 | SKI | 6497 |
| 2 | MAD2L2 | 10459 |
| 3 | SDHB | 6390 |
| 4 | MYCL1 | 4610 |
| 5 | MPL | 4352 |
| 6 | PLK3 | 1263 |
| 7 | MUTYH | 4595 |
| 8 | CDKN2C | 1031 |
| 9 | BCL10 | 8915 |
| 10 | NRAS | 4893 |
| 11 | NGF | 4803 |
| 12 | PRCC | 5546 |
| 13 | NTRK1 | 4914 |
| 14 | SDHC | 6391 |
| 15 | FH | 2271 |
| 16 | MYCN | 4613 |
| 17 | CENPA | 1058 |
| 18 | ALK | 238 |
| 19 | EML4 | 27436 |
| 20 | MSH2 | 4436 |
| 21 | MSH6 | 2956 |
| 22 | REL | 5966 |
| 23 | BUB1 | 699 |
| 24 | ERCC3 | 2071 |
| 25 | COL3A1 | 1281 |
| 26 | COL5A2 | 1290 |
| 27 | PMS1 | 5378 |
| 28 | CREB1 | 1385 |
| 29 | IDH1 | 3417 |
| 30 | ERBB4 | 2066 |
| 31 | PAX3 | 5077 |
| 32 | CUL3 | 8452 |
| 33 | COL4A4 | 1286 |
| 34 | COL4A3 | 1285 |
| 35 | COL6A3 | 1293 |
| 36 | FANCD2 | 2177 |
| 37 | VHL | 7428 |
| 38 | RAF1 | 5894 |
| 39 | XPC | 7508 |
| 40 | TGFBR2 | 7048 |
| 41 | MLH1 | 4292 |
| 42 | CTNNB1 | 1499 |
| 43 | MITF | 4286 |
| 44 | GATA2 | 2624 |
| 45 | AC128683.3 | 131873 |

| **Nr.** | **Associated Gene Name** | **Entrez Gene ID** |
| --- | --- | --- |
| 46 | PIK3CA | 5290 |
| 47 | ETV5 | 2119 |
| 48 | BCL6 | 604 |
| 49 | FGFR3 | 2261 |
| 50 | RHOH | 399 |
| 51 | PHOX2B | 8929 |
| 52 | PDGFRA | 5156 |
| 53 | KIT | 3815 |
| 54 | KDR | 3791 |
| 55 | CENPE | 1062 |
| 56 | MAD2L1 | 4085 |
| 57 | FBXW7 | 55294 |
| 58 | LIFR | 3977 |
| 59 | IL6ST | 3572 |
| 60 | PLK2 | 10769 |
| 61 | PIK3R1 | 5295 |
| 62 | APC | 324 |
| 63 | ARHGAP26 | 23092 |
| 64 | PDGFRB | 5159 |
| 65 | NPM1 | 4869 |
| 66 | FLT4 | 2324 |
| 67 | IRF4 | 3662 |
| 68 | HIST1H1B | 3009 |
| 69 | FANCE | 2178 |
| 70 | CDKN1A | 1026 |
| 71 | CCND3 | 896 |
| 72 | ROS1 | 6098 |
| 73 | GOPC | 57120 |
| 74 | CARD11 | 84433 |
| 75 | PMS2 | 5395 |
| 76 | ETV1 | 2115 |
| 77 | JAZF1 | 221895 |
| 78 | RALA | 5898 |
| 79 | IKZF1 | 10320 |
| 80 | EGFR | 1956 |
| 81 | SBDS | 51119 |
| 82 | CDK6 | 1021 |
| 83 | COL1A2 | 1278 |
| 84 | MET | 4233 |
| 85 | SMO | 6608 |
| 86 | KIAA1549 | 57670 |
| 87 | BRAF | 673 |
| 88 | PCM1 | 5108 |
| 89 | WRN | 7486 |
| 90 | FGFR1 | 2260 |

| **Nr.** | **Associated Gene Name** | **Entrez Gene ID** |
| --- | --- | --- |
| 91 | MYST3 | 7994 |
| 92 | NBN | 4683 |
| 93 | EXT1 | 2131 |
| 94 | MYC | 4609 |
| 95 | RECQL4 | 9401 |
| 96 | JAK2 | 3717 |
| 97 | CDKN2A | 1029 |
| 98 | CDKN2B | 1030 |
| 99 | FANCG | 2189 |
| 100 | PAX5 | 5079 |
| 101 | GNAQ | 2776 |
| 102 | FANCC | 2176 |
| 103 | PTCH1 | 5727 |
| 104 | XPA | 7507 |
| 105 | TGFBR1 | 7046 |
| 106 | ABL1 | 25 |
| 107 | TSC1 | 7248 |
| 108 | COL5A1 | 1289 |
| 109 | NOTCH1 | 4851 |
| 110 | COPEB | 1316 |
| 111 | RET | 5979 |
| 112 | NCOA4 | 8031 |
| 113 | BMPR1A | 657 |
| 114 | PTEN | 5728 |
| 115 | FAS | 355 |
| 116 | NFKB2 | 4791 |
| 117 | SUFU | 51684 |
| 118 | FGFR2 | 2263 |
| 119 | HRAS | 3265 |
| 120 | CDKN1C | 1028 |
| 121 | FANCF | 2188 |
| 122 | WT1 | 7490 |
| 123 | EXT2 | 2132 |
| 124 | DDB2 | 1643 |
| 125 | INCENP | 3619 |
| 126 | MEN1 | 4221 |
| 127 | CCND1 | 595 |
| 128 | BIRC3 | 330 |
| 129 | ATM | 472 |
| 130 | SDHD | 6392 |
| 131 | MLL | 4297 |
| 132 | ARHGEF12 | 23365 |
| 133 | CCND2 | 894 |
| 134 | ETV6 | 2120 |
| 135 | CDKN1B | 1027 |
| 136 | KRAS | 3845 |
| 137 | COL2A1 | 1280 |

| **Nr.** | **Associated Gene Name** | **Entrez Gene ID** |
| --- | --- | --- |
| 138 | ATF1 | 466 |
| 139 | ERBB3 | 2065 |
| 140 | CDK4 | 1019 |
| 141 | MDM2 | 4193 |
| 142 | BTG1 | 694 |
| 143 | PTPN11 | 5781 |
| 144 | HNF1A | 6927 |
| 145 | FLT3 | 2322 |
| 146 | FLT1 | 2321 |
| 147 | BRCA2 | 675 |
| 148 | RB1 | 5925 |
| 149 | ERCC5 | 2073 |
| 150 | COL4A1 | 1282 |
| 151 | COL4A2 | 1284 |
| 152 | CDKN3 | 1033 |
| 153 | MLH3 | 27030 |
| 154 | TSHR | 7253 |
| 155 | AKT1 | 207 |
| 156 | BUB1B | 701 |
| 157 | SMAD3 | 4088 |
| 158 | NTRK3 | 4916 |
| 159 | BLM | 641 |
| 160 | TSC2 | 7249 |
| 161 | CREBBP | 1387 |
| 162 | ERCC4 | 2072 |
| 163 | PALB2 | 79728 |
| 164 | PLK1 | 5347 |
| 165 | FUS | 2521 |
| 166 | CYLD | 1540 |
| 167 | CDH11 | 1009 |
| 168 | CDH1 | 999 |
| 169 | FANCA | 2175 |
| 170 | TP53 | 7157 |
| 171 | AURKB | 9212 |
| 172 | MAP2K4 | 6416 |
| 173 | FLCN | 201163 |
| 174 | NF1 | 4763 |
| 175 | SUZ12 | 23512 |
| 176 | ERBB2 | 2064 |
| 177 | RARA | 5914 |
| 178 | BRCA1 | 672 |
| 179 | ETV4 | 2118 |
| 180 | COL1A1 | 1277 |
| 181 | BRIP1 | 83990 |
| 182 | PRKAR1A | 5573 |
| 183 | KIAA1618 | 57714 |
| 184 | ASPSCR1 | 79058 |

| **Nr.** | **Associated Gene Name** | **Entrez Gene ID** |
| --- | --- | --- |
| 185 | SMAD2 | 4087 |
| 186 | SMAD4 | 4089 |
| 187 | MALT1 | 10892 |
| 188 | BCL2 | 596 |
| 189 | STK11 | 6794 |
| 190 | COL5A3 | 50509 |
| 191 | CDKN2D | 1032 |
| 192 | BRD4 | 23476 |
| 193 | JAK3 | 3718 |
| 194 | CEBPA | 1050 |
| 195 | AKT2 | 208 |
| 196 | ERCC2 | 2068 |
| 197 | BAX | 581 |
| 198 | AURKC | 6795 |
| 199 | CENPB | 1059 |
| 200 | GNAS | 2778 |
| 201 | GNAS | 2778 |
| 202 | RUNX1 | 861 |
| 203 | ERG | 2078 |
| 204 | COL6A1 | 1291 |
| 205 | COL6A2 | 1292 |
| 206 | SMARCB1 | 6598 |
| 207 | CHEK2 | 11200 |
| 208 | EWSR1 | 2130 |
| 209 | NF2 | 4771 |
| 210 | PDGFB | 5155 |
| 211 | EP300 | 2033 |
| 212 | ARAF | 369 |
| 213 | WAS | 7454 |
| 214 | GATA1 | 2623 |
| 215 | FAM123B | 139285 |
| 216 | ERCC6L | 54821 |
| 217 | COL4A6 | 1288 |
| 218 | COL4A5 | 1287 |
| 219 | GPC3 | 2719 |

**Supplemental Table 2a:** HighConfidence variants filtered from the HCDiff variant table for P-TU. The table includes chromosome ID, relative start and end position of the variant (reference genome: hg18 and SNPdb built 130), reference and variant nucleotide, sequencing depth and variation frequency at the respective position, reference and variant amino-acid as well as variation type (non-synonymous/synonymous) and the validated status (germline/somatic origin) of the variant. Variants that could not be confirmed by Sanger Sequencing are denoted “false positives”.


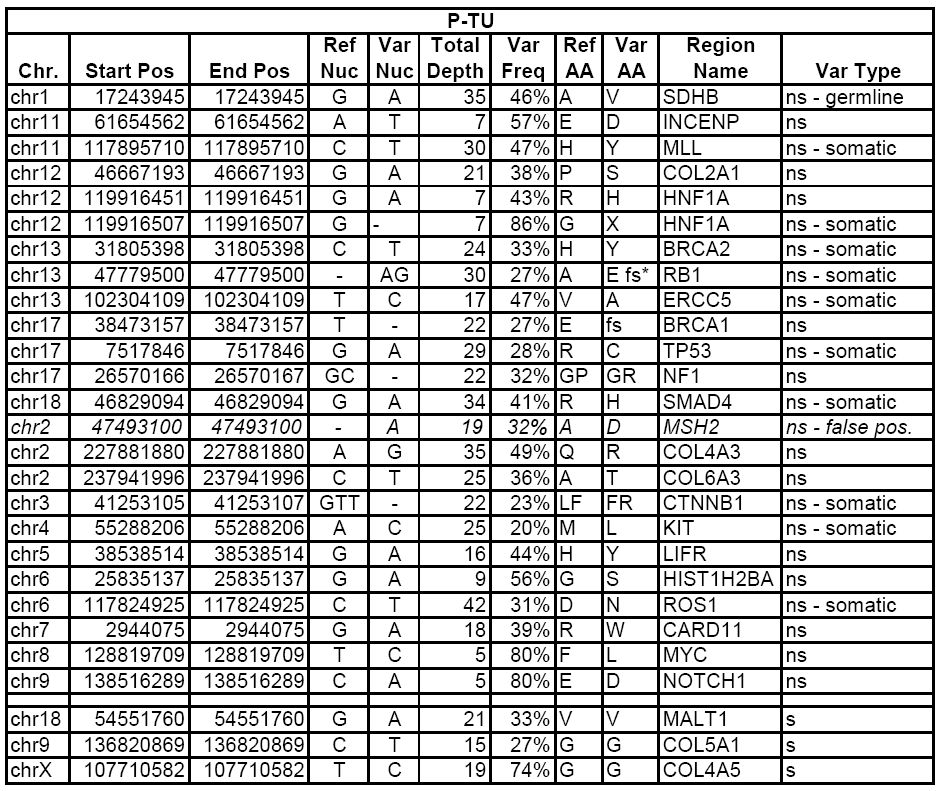


**Supplemental Table 2b:** HighConfidence variants filtered from the HCDiff variant table for P-STS P41.


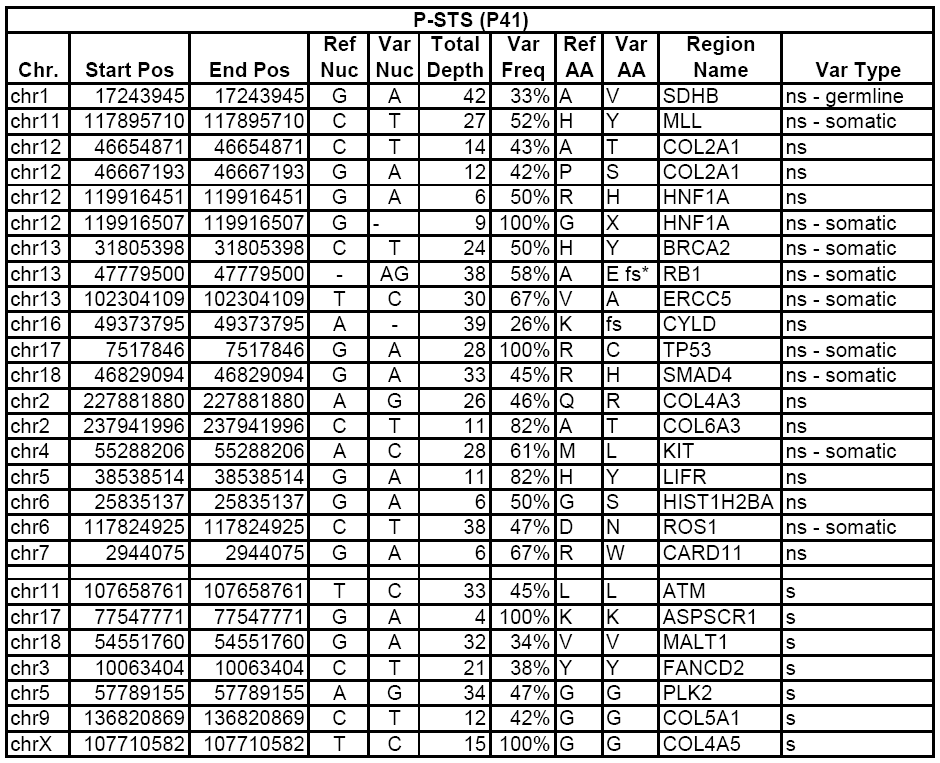


**Supplemental Table 2c and d:** HighConfidence variants filtered from the HCDiff variant table for H-STS P42 (c) and L-STS P49 (d).

b)


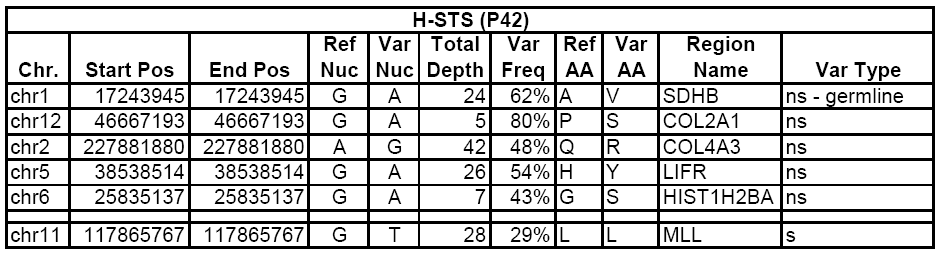


c)


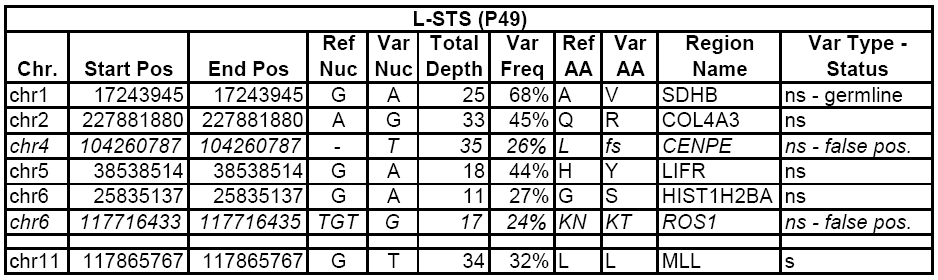


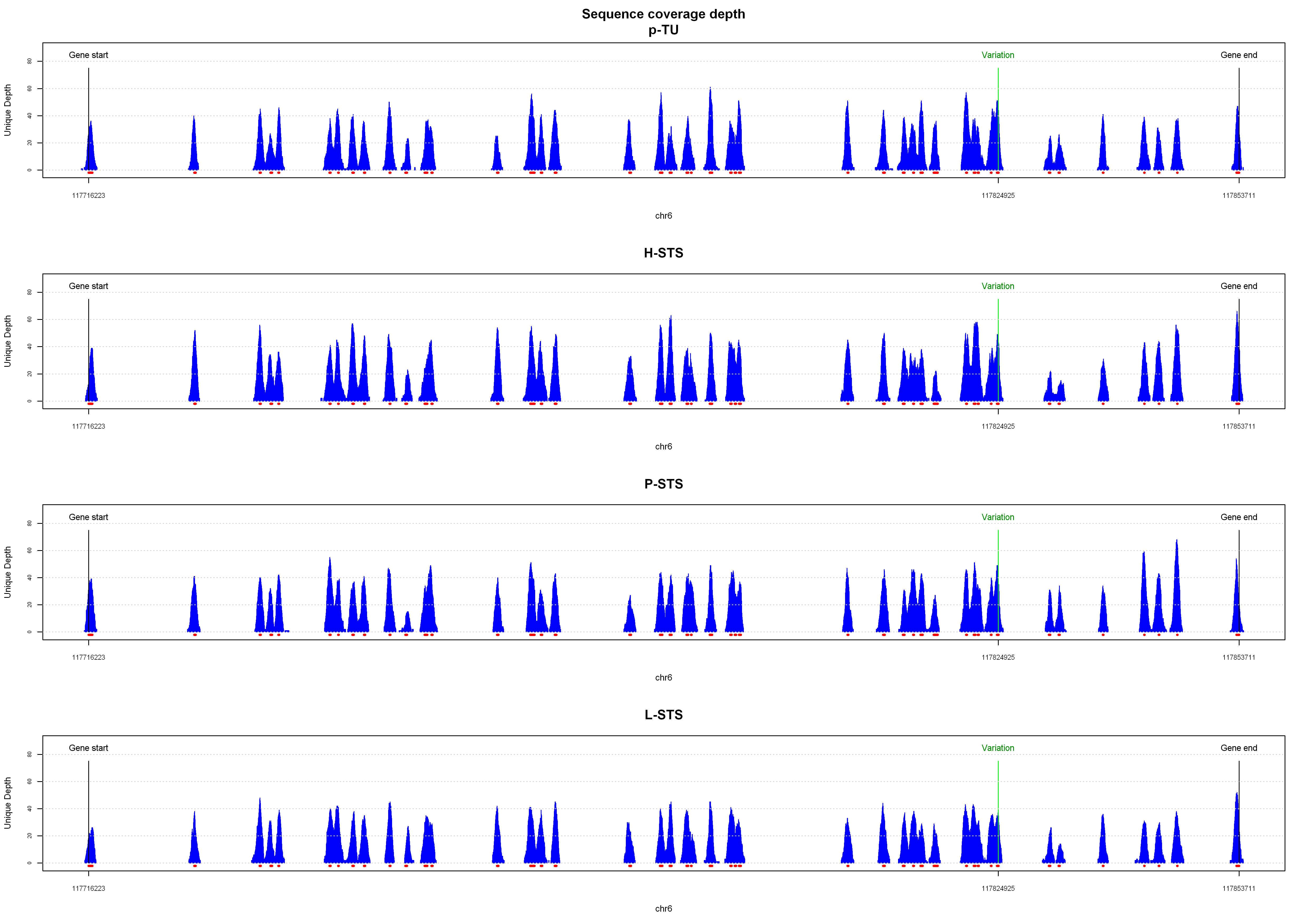
**Supplemental Figure 1.** Graphical representation of comparative sequence coverage unique depth (y-axis: number of non-duplicate, uniquely mapped reads) for a representative locus (*BRCA2*) with the chromosomal positions (x-axis) of the detected sequence variation marked in green and exonic regions marked red. Each sample is represented in a separate diagram.

**Supplemental Figure 2:** High Resolution Melt Analysis. To detect sequence variants with higher sensitivity compared to Sanger Sequencing, HRM analysis was performed for the *RB1* and *MLL* variant in P-TU, P-STS P41, L-STS P49 and H-STS P42. **(A)** Normalized and temperature shifted difference plots for the RB1 amplicon carrying the ins AG211-212 mutation site. Sanger sequencing chromatograms show a heterozygote AG211-213 ins in P-TU and P-STS P41 samples compared to H-STS P42 and L-STS P49. **(B)** Normalized and temperature shifted difference plots for the MLL amplicon comprising the c11305 position. Sanger Sequencing reveals cC11305T heterozygosity in P-TU and P-STS P41 samples whereas H-STS P42 and L-STS P49 show cC11305 homozygosity. **C)** A standard curve generated by mixing genomic DNAs derived from heterozygote P-TU and homozygote late passage of L-STS (representing 50%, 25%, 16.7%, 12,5% and 10% of the variant allele) revealed an analytical sensitivity of ~10% for detection of the RB1 (left) and MLL (right) variant.

A

B


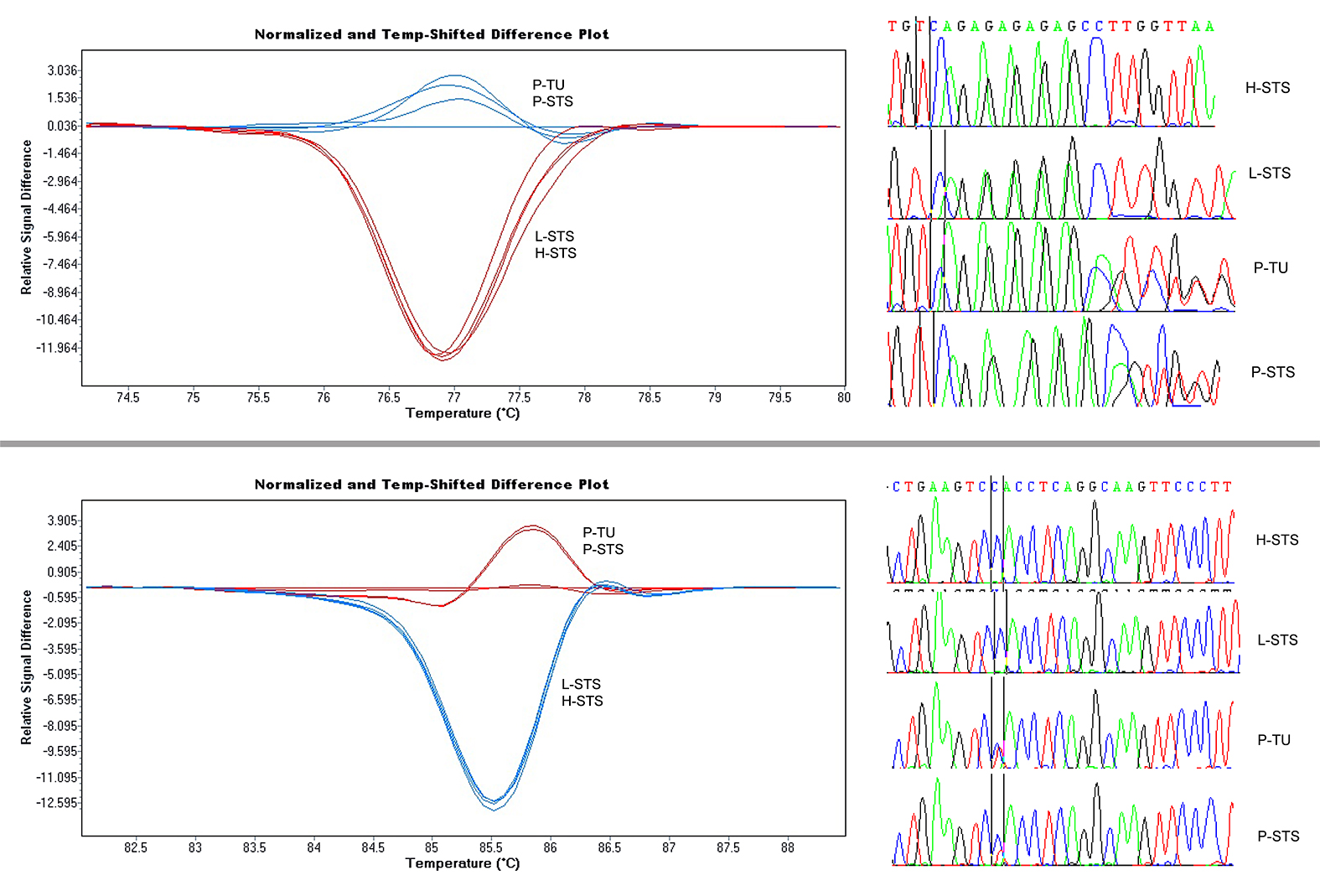


A

B


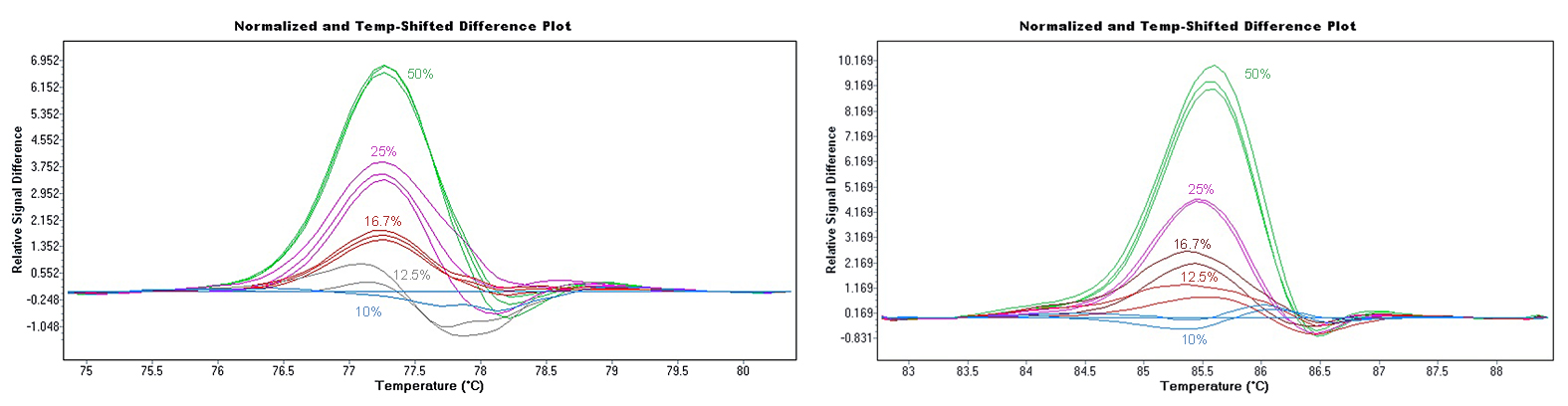


C

RB1

MLL

**Supplemental Table 3:** Primer Sequences for Sanger Sequencing, High Resolution Melting Curve Analysis and quantitative Real-time PCR.

| **Genesymbol** | **AccNr** | **Primer** | **Sequence 5'-3'** |
| --- | --- | --- | --- |
| RB1 | NM_000321.1 | Seq_RB1_ex2_fw | TCACTGTGTGGTATCCTTATTTTG |
|  |  | Seq_RB1_ex2_rev | CCGGCCTCAAACATTTTTA |
|  |  | HRM_RB1_fw | TGGTAGGCTTGAGTTTGAAGAA |
|  |  | HRM_RB1_rev | CCAATACTCCATCCACAGATGA |
|  |  | qPCR_RB1_fw | ATGGCATATGCAAAGTGAAGA |
|  |  | qPCR_RB1_rev | GGGGGCCTGGTGGAAGCATA |
| CTNNB1 | NM_001098209.1 | Seq_CTNNB1_ex13_fw | AGGACAAGGAAGCTGCAGAA |
|  |  | Seq_CTNNB1_ex13_rev | GCTGCCATACCTGCTCTAGG |
| MLL | NM_005933.2 | Seq_MLL_ex32_fw | CCATGATGCAGTTGTGTTCC |
|  |  | Seq_MLL_ex32_rev | ATCGAGACCCAAAACTGCTG |
|  |  | HRM_MLL_fw | CCATGATGCAGTTGTGTTCC |
|  |  | HRM_MLL_rev | ATCGAGACCCAAAACTGCTG |
|  |  | qPCR_MLL_fw | CTTCCTGGCTTCTAAACATCGTCA |
|  |  | qPCR_MLL_rev | TGCCGGCATACTCAATCACC |
| BRCA2 | NM_000059.3 | Seq_BRCA2_ex9_fw | GCCTCTGAAAGTGGACTGGA |
|  |  | Seq_BRCA2_ex9_rev | GCTTCAAACTGGGCTGAACA |
|  |  | qPCR_BRCA2_fw | GGCCAAGTTCCCTCTGCGTGTTC |
|  |  | qPCR_BRCA2_rev | TCTTTTCCAGCCTTTCCATCATTG |
| ERCC5 | NM_000123.2 | Seq_ERCC5_ex3_fw | TGGCAATTAGGAGGAAATGC |
|  |  | Seq_ERCC5_ex3_rev | AAGGCAGTTTTGATGGCTTG |
|  |  | qPCR_ERCC5_fw | CTCCGGGCGGCAGGTCAG |
|  |  | qPCR_ERCC5_rev | GAGCATCCCCATCAAACACAAAAA |
| KIT | NM_000222.2 | Seq_KIT_ex10_fw | CCACACCCTGTTCACTCCTT |
|  |  | Seq_KIT_ex10_rev | GTGGGGAGAAAGGGAAAAAT |
|  |  | qPCR_KIT_fw | CCACCAACACCGGCAAATACAC |
|  |  | qPCR_KIT_rev | CCCATACAAGGAGCGGTCAACAAG |
| SMAD4 | NM_005359.5 | Seq_SMAD4_ex2 | CGGAAAGGATTTCCTCATGT |
|  |  | Seq_SMAD4_ex2 | TAAAGTCGCGGGCTATCTTC |
|  |  | qPCR_SMAD4_fw | CTCAGCCAGGACAGCAGCAGAATG |
|  |  | qPCR_SMAD4_rev | TGAAGATGGCCGTTTTGGTGGTGA |
| ROS1 | NM_002944.2 | Seq_ROS1_ex7_fw | GGAAAGGAGCGAACTGGTTT |
|  |  | Seq_ROS1_ex7_rev | GGTTCTCTGTGTCCCTGCAT |
|  |  | qPCR_ROS1_fw | CAGCAGTTGACCTTGGCACAC |
|  |  | qPCR_ROS1_rev | AACCTCACACGACTCCCGACACTT |
| HNF1A1 | NM_000545.4 | Seq-HNF1A1_ex4_fw | TGTCAATTGCCCAAGGTCA |
|  |  | Seq-HNF1A1_ex4_fw | TGGAATGGATGAATGAACAGAG |
|  |  | qPCR_HNF1A_fw | GAGAGGTGGCGGCAGCAGTTCA |
|  |  | qPCR_HNF1A_fw | CTCCGCCCCTTCTTGGTTGGTA |
| TP53 | NM_000546.4 | Seq-TP53_ex4_fw | CCTGGTCCTCTGACTGCTCTTTTCACCCA |
|  |  | Seq-TP53_ex4_rev | GGCCAGGCATTGAAGTCTCAT |
|  |  | Seq-TP53_ex7_fw | CCTCATCTTGGGCCTGTGTT |
|  |  | Seq-TP53_ex7_rev | AGTGTGCAGGGTGGCAAGTG |
|  |  | Seq-TP53_ex8_fw | CAAGGGTGGTTGGGAGTAGA |
|  |  | Seq-TP53_ex8_rev | TAACTGCACCCTTGGTCTCC |
|  |  | qPCR_TP53_fw | CCCTCCTGGCCCCGCTCATCT |
|  |  | qPCR_TP53_rev | GGCGGGGGTGTGGAATCAAC |

**Supplemental Table 4:** Summary of CNV/Genotype data for primary tumor tissue P-TU and the metastatic tissues (H-Met and L-Met), as well as data for the associated cell lines P-STS P41, H-STS P42, and L-STS P49. The Copy neutral LOH is conserved in the primary tumor and the metastatic tissues. The P-STS –P41 cell line harbours the LOH at chromosome 3 combined with a loss of the second allele (CN state 1). The P-STS cell line has gained additional chromosomal alterations during cultivation, whereas the metastases derived cell lines (H-STS –P42 and L-STS –P49) display a normal chromosomal status using Affymetrix 6.0 CNV/GT SNP microarrays.


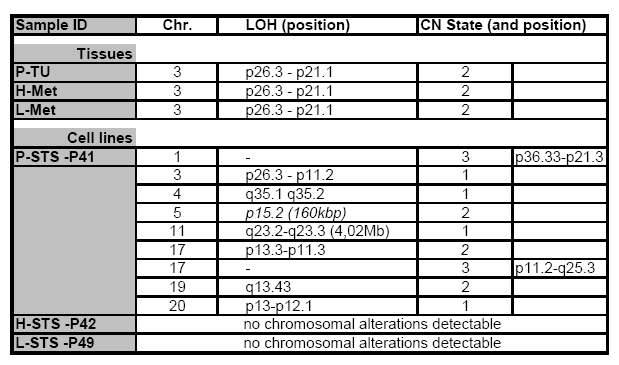


**Supplemental Figure 3:** CNV analysis of the P-STS. CN changes were detectable for chromosomes 1, 3, 4, 11, 17 and 20 and are summarized in supplemental information table 4.


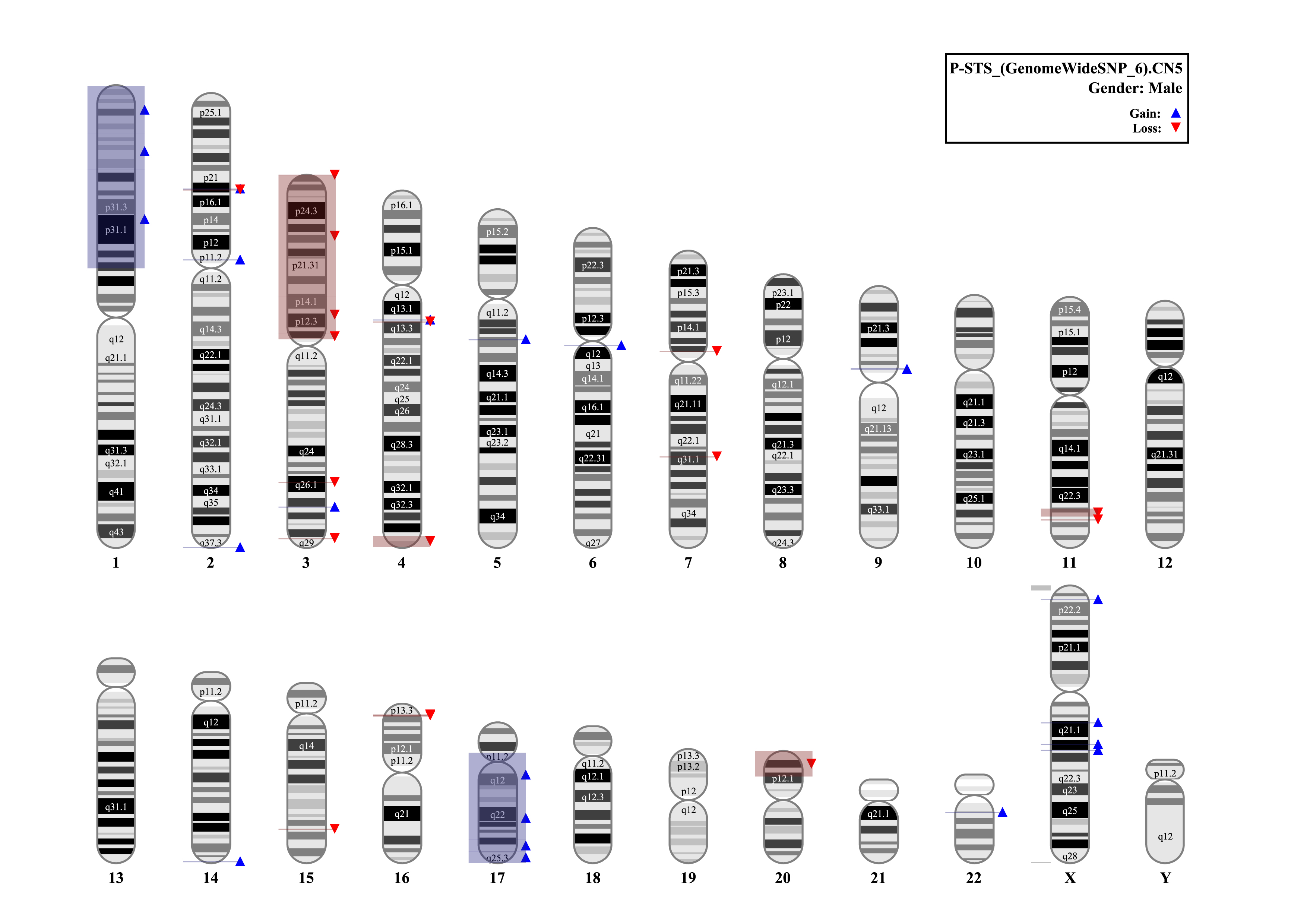


**Supplemental Figure 4a:** CNV and genotype data generated for the *MLL* locus. No CNV was detected in any of the four samples.


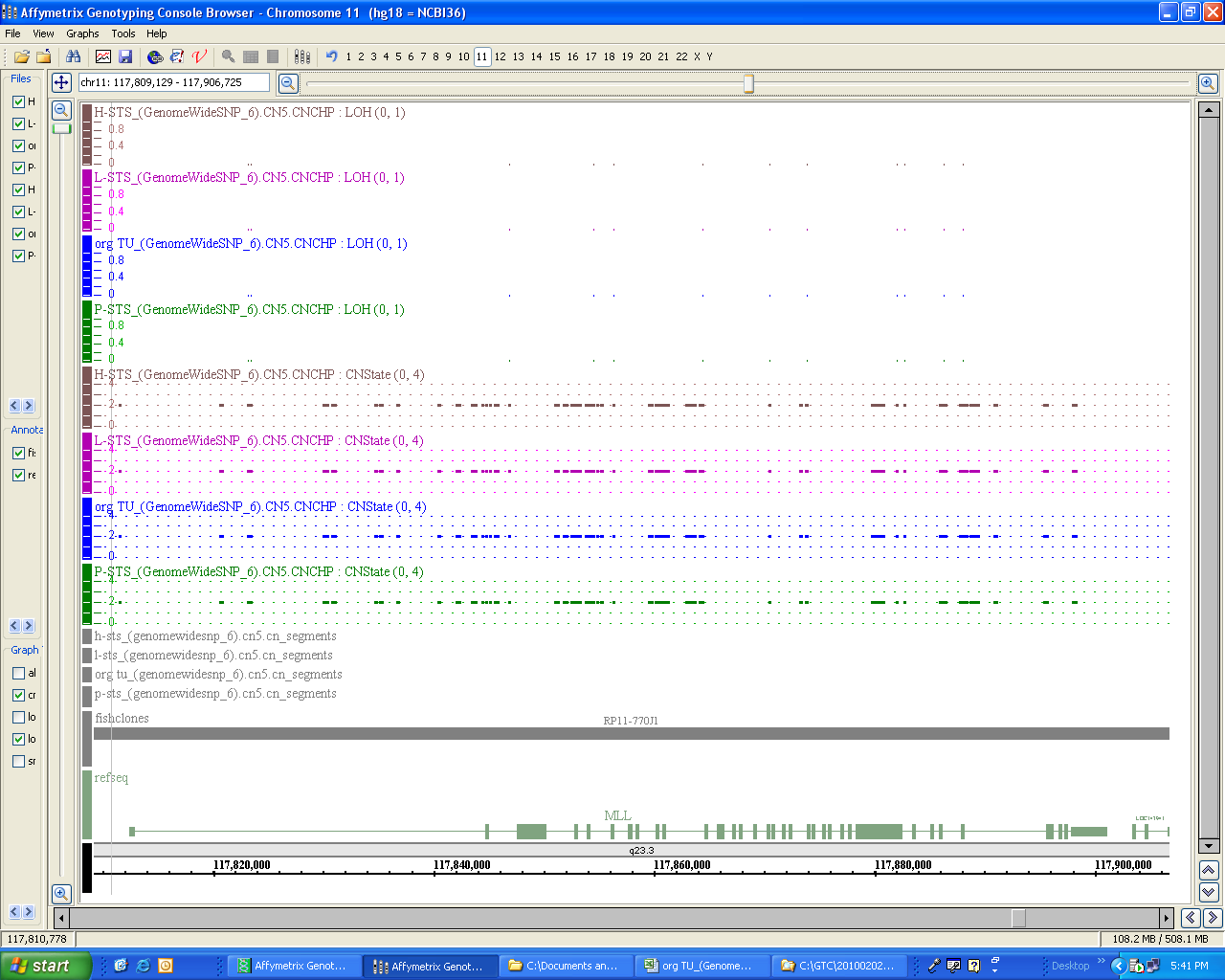


**Supplemental Figure 4b:**: CNV and genotype data generated for the *BRCA2*  locus. No CNV was detected in any of the four samples.


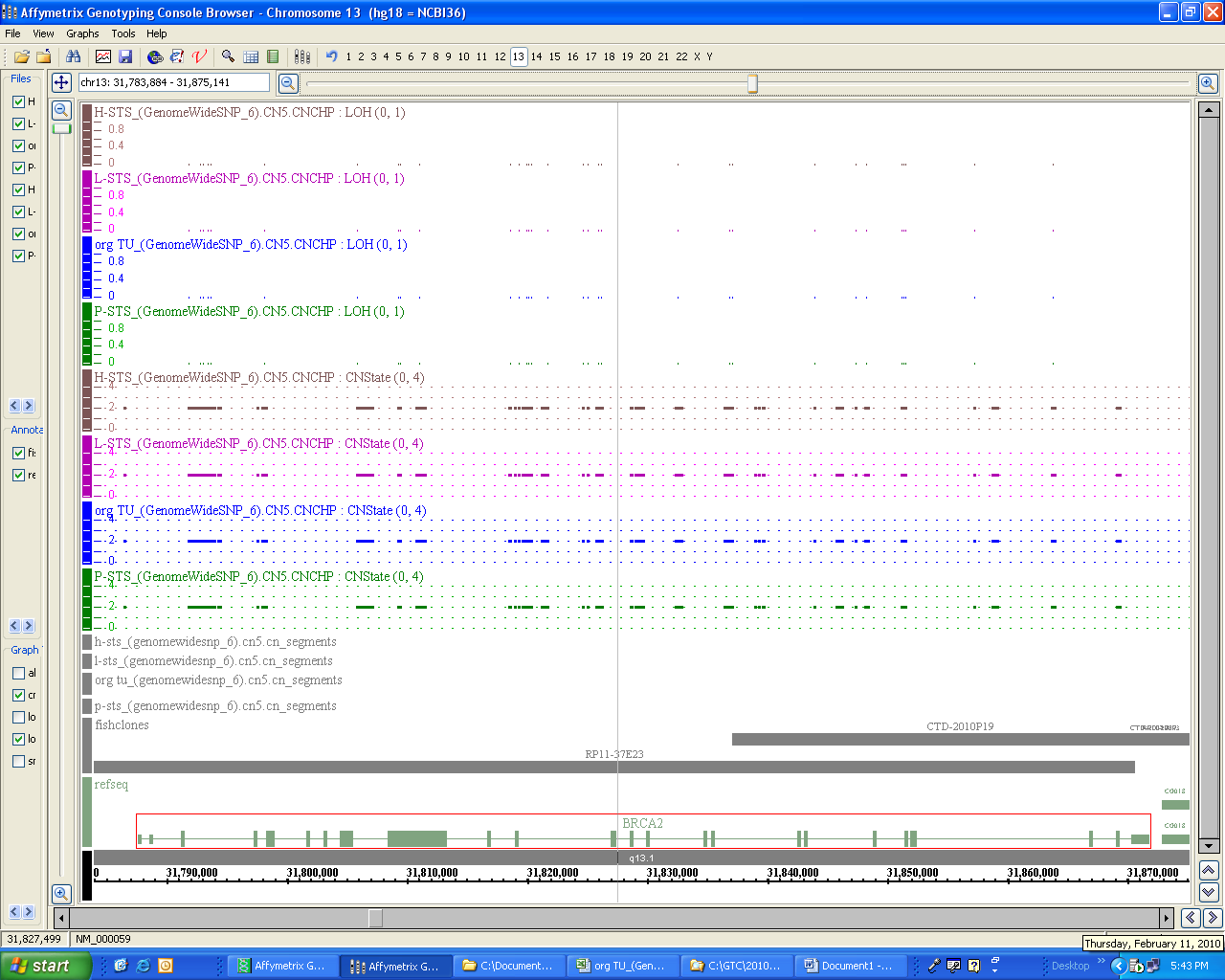


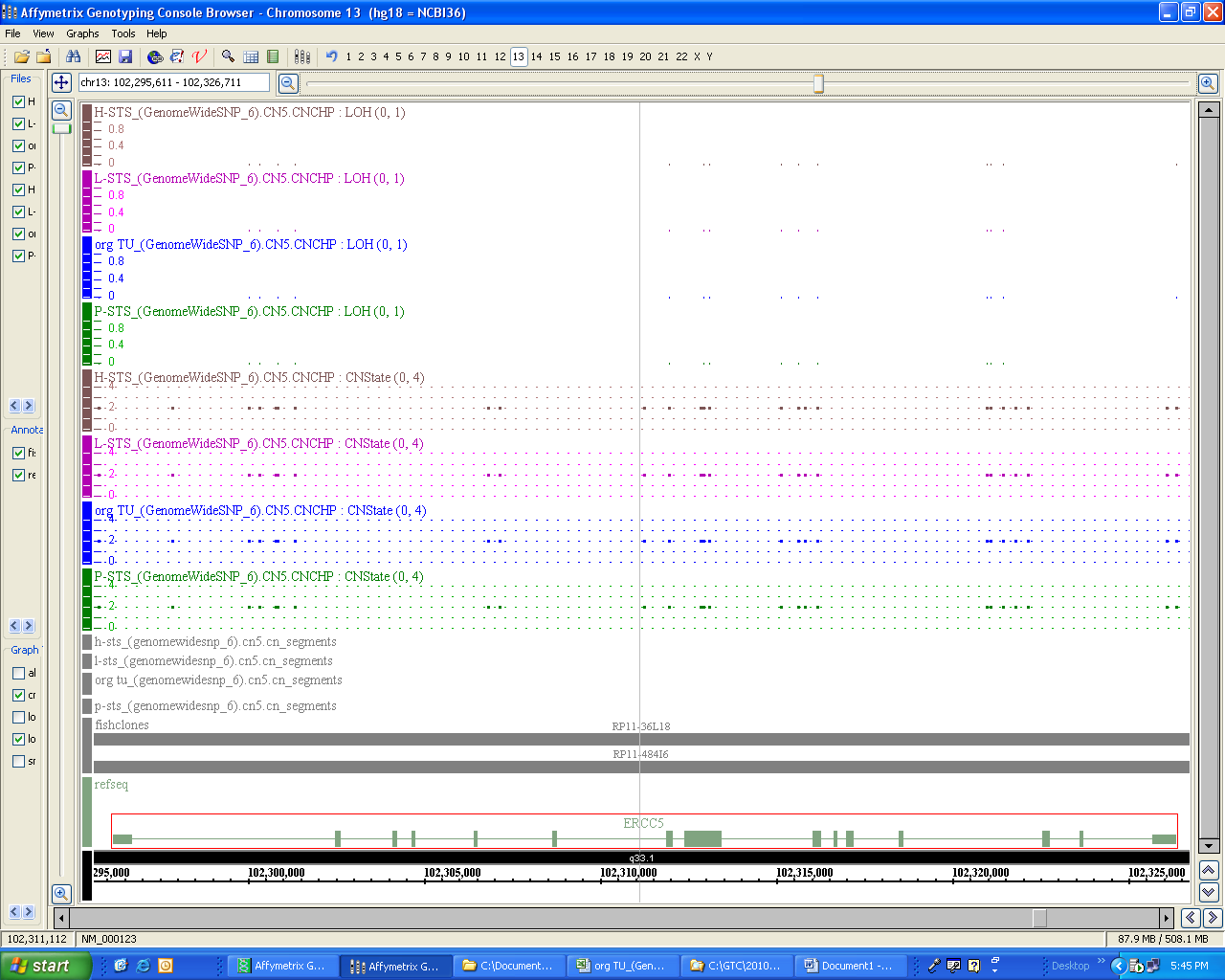
**Supplemental Figure 4c:**: CNV and genotype data generated for the *ERCC5*  locus. No CNV was detected in any of the four samples.

**Supplemental Figure 4d:** CNV and genotype data generated for the *KIT*  locus. No CNV was detected in any of the four samples.


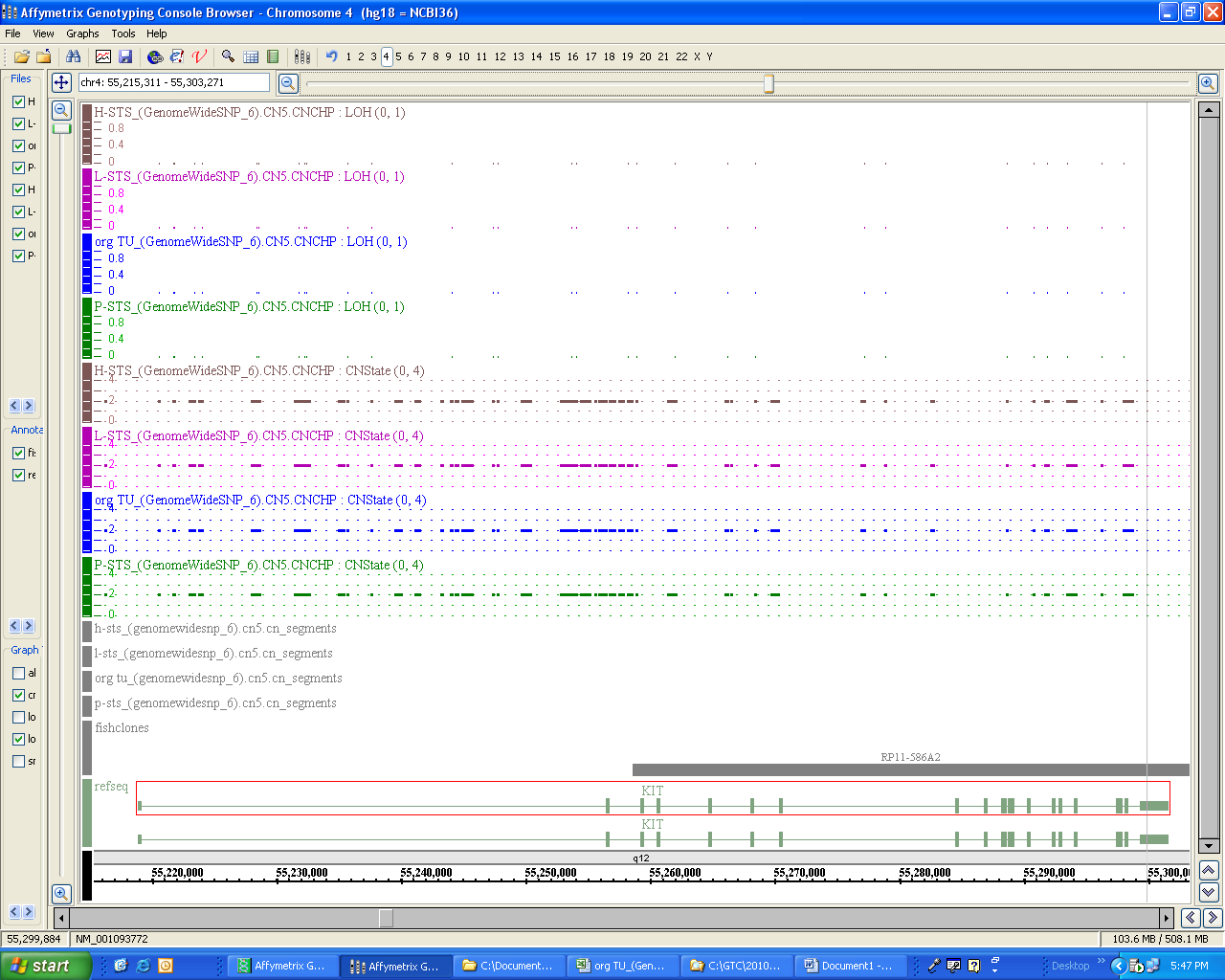


**Supplemental Figure 4e:** CNV and genotype data generated for the *TP53*  locus. No CNV was detected in any of the four samples. Genotype data for the P-STS sample indicate lack of heterozygosity.


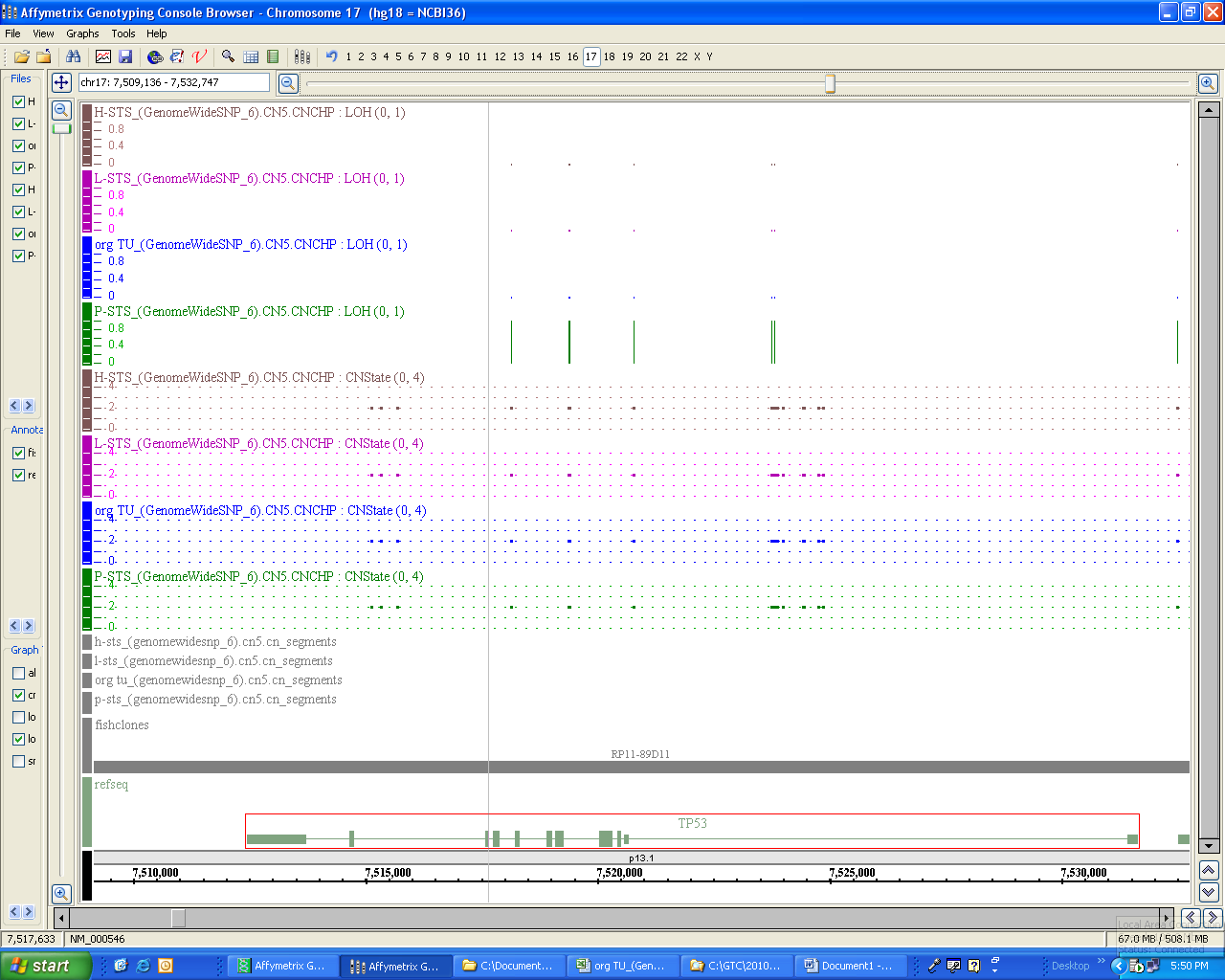


**Supplemental Figure 4f:** CNV and genotype data generated for the *ROS1*  locus. No CNV was detected in any of the four samples.


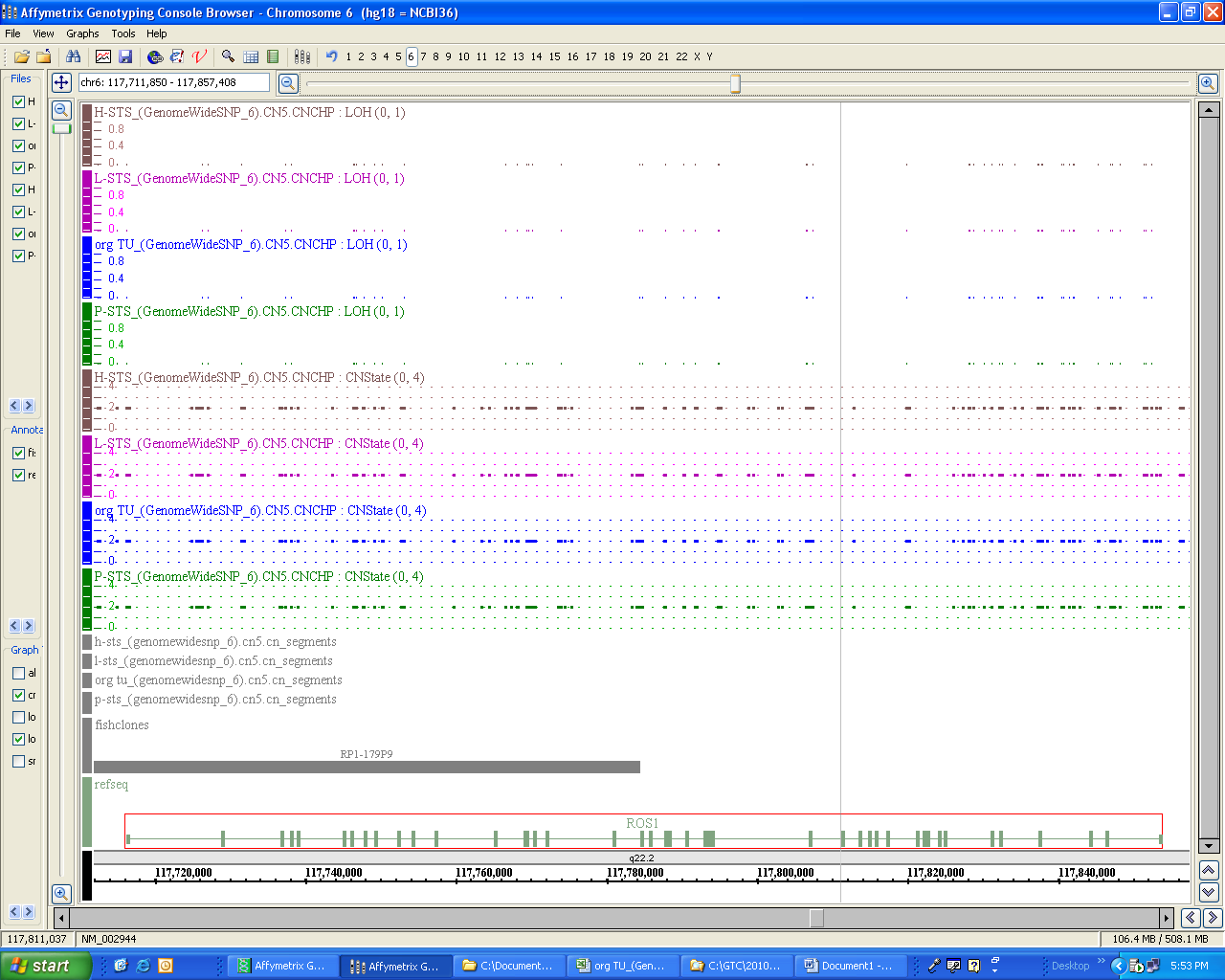


**Supplemental Figure 4g:** CNV and genotype data generated for the *HNF1A*  locus. No CNV was detected in any of the four samples.


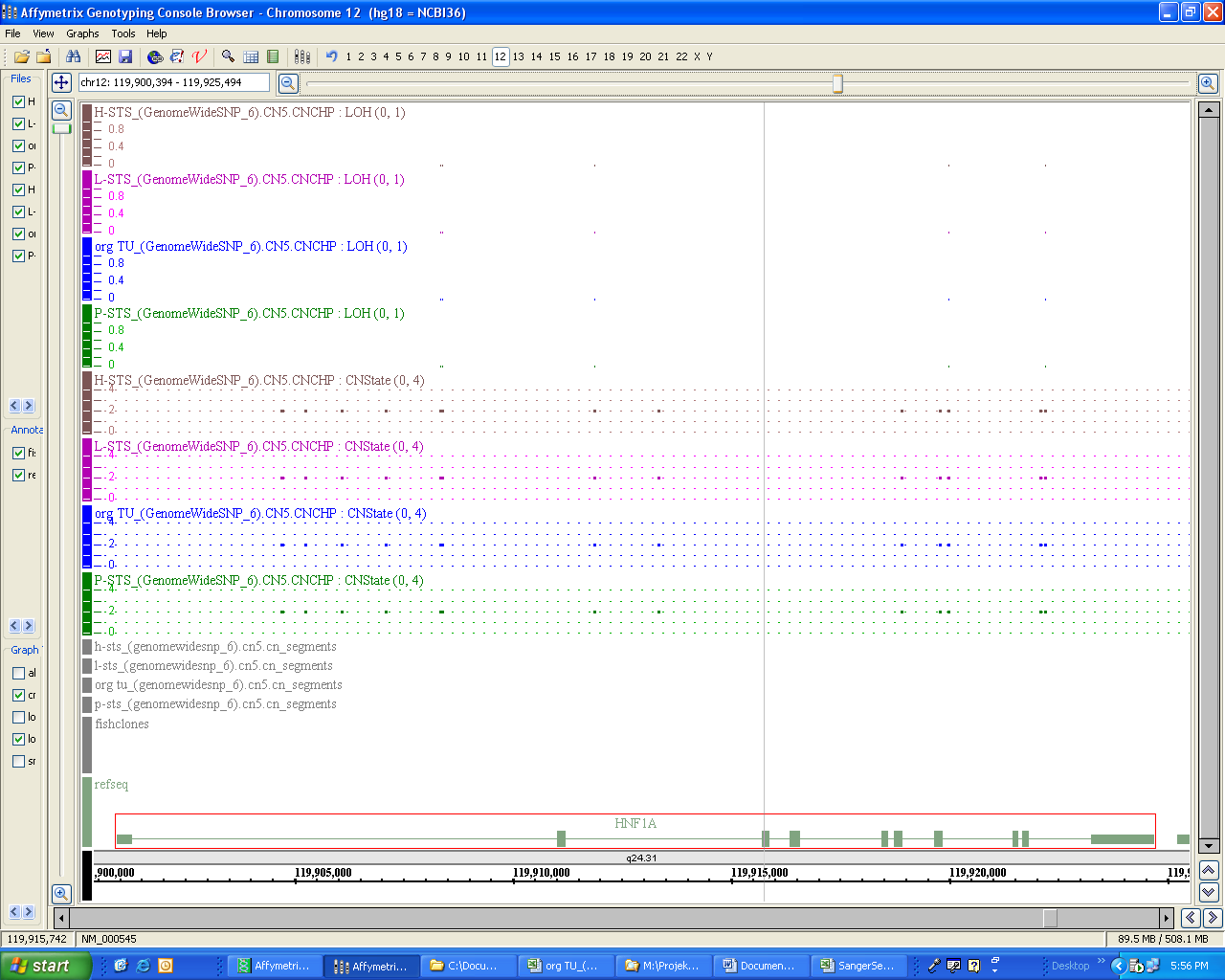


**Supplemental Figure 4h:** CNV and genotype data generated for the *SMAD4*  locus. No CNV was detected in any of the four samples.


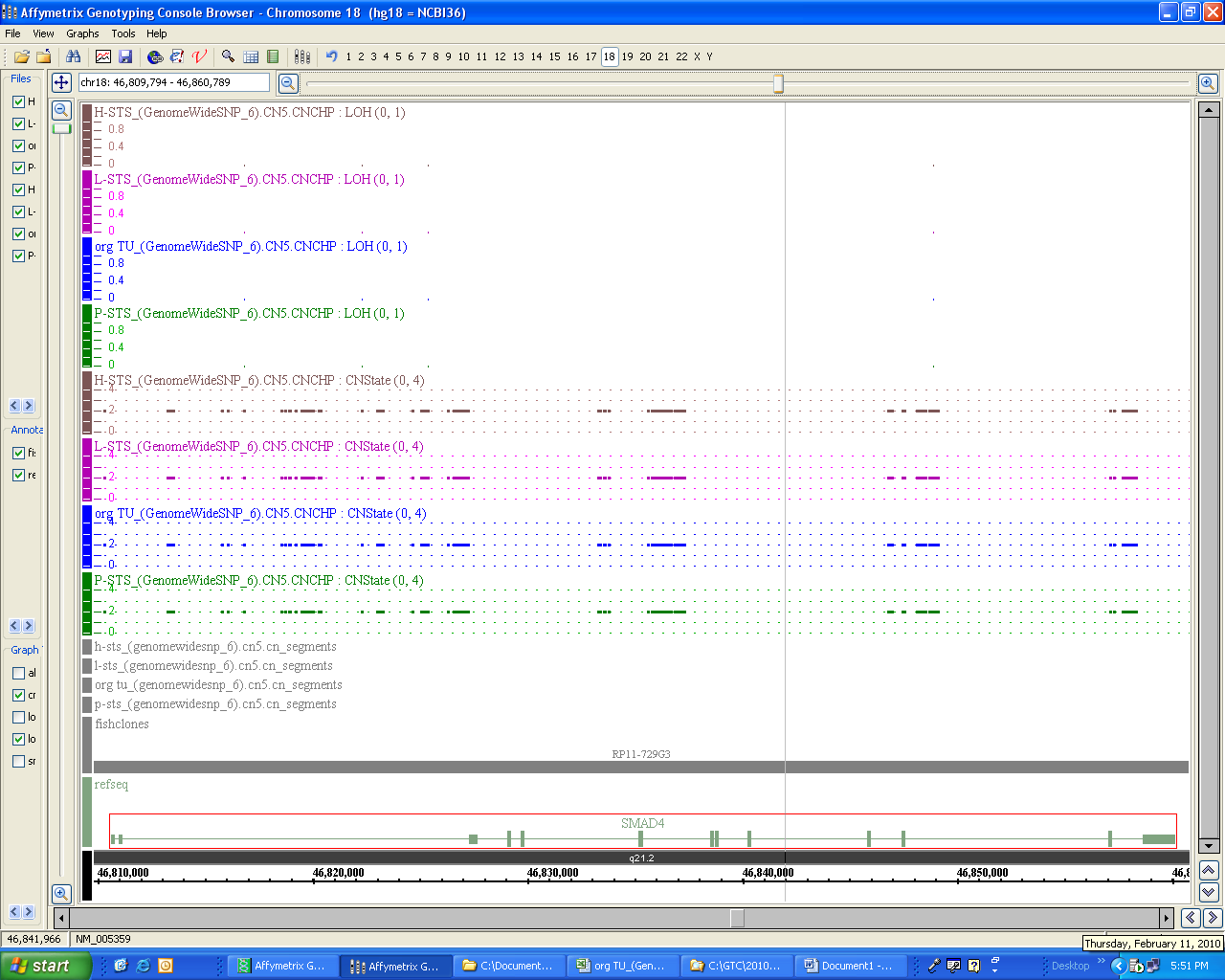


**Supplemental Figure 4i:** CNV and genotype data generated for the *RB1*  locus. No CNV was detected in any of the four samples.


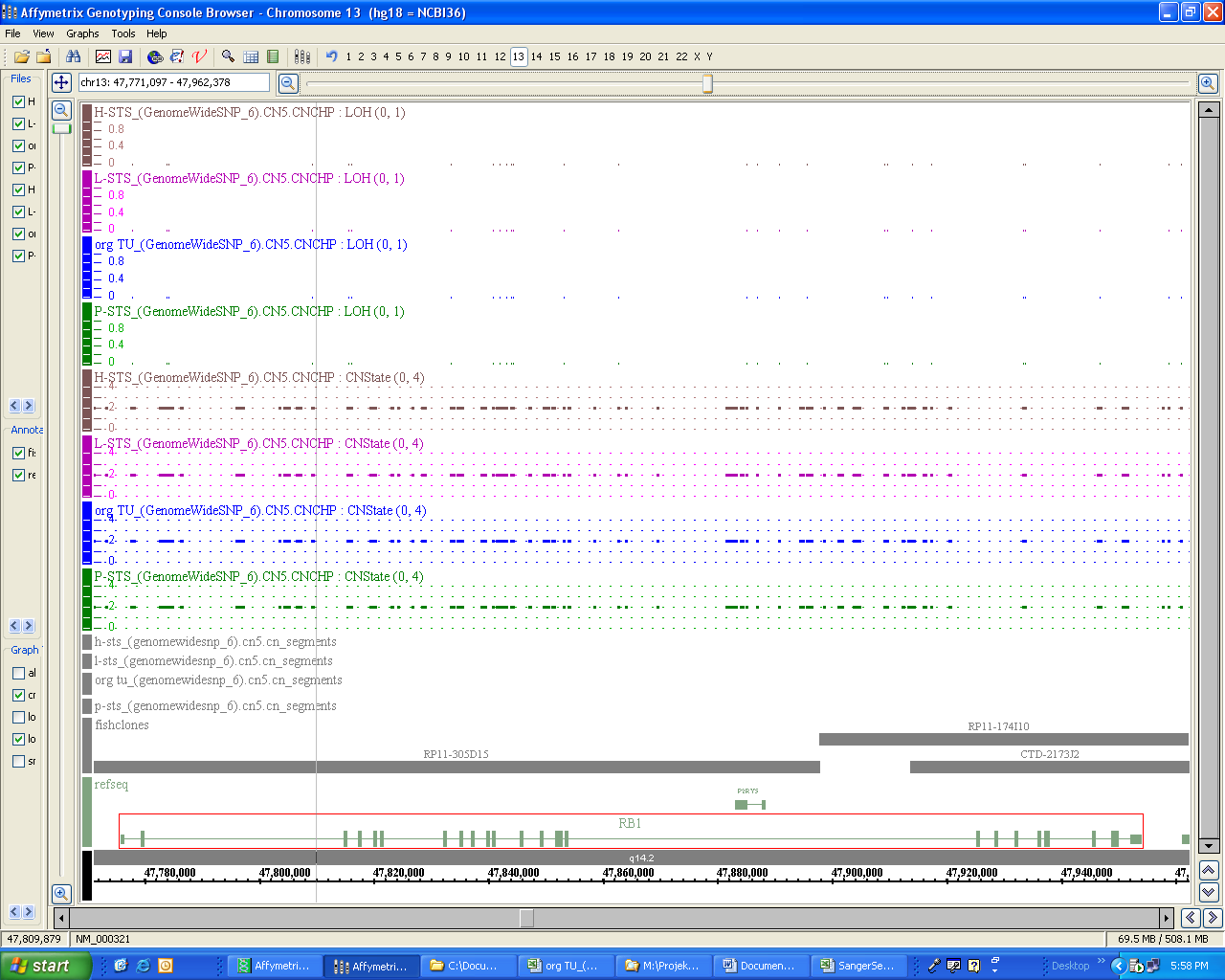


**Supplemental Experimental Procedures**

**Supplemental Experimental Procedure 1:**

**Sequence Capture Library Construction**

Genomic DNA isolated from the snap-frozen primary tumor sample and the three corresponding cell lines (P-STS, H-STS and L-STS) was processed into a capture library. 20µg of genomic DNA was fragmented by nebulisation of the DNA (at 3bar N_2_ for one minute) in an appropriate nebulisation device (Nimblegen). An average DNA strand length of approximately 550bp was determined by using capillary electrophoresis on a DNA7500 chip in the BioAnalyzer 2100 (Agilent, Palo Alto, CA). The genomic DNA was polished and repaired using a combined T4 DNA polymerase (3U/µl) and T4 PNK (10U/µl) (New England Biolabs, Ipswich, MA) in a 120-µl volume. The polishing master mix contained 12 µl 10 x NEB T4 polymerase buffer (NEB2), 9 µl water, 1 µl 100 x NEB BSA, 5 µl 25 mM dNTP mix (dATP, dTTP, dCTP, dGTP) (Roche, Mannheim, D), 1 µl 100 mM rATP, 6 µl (18 U) NEB T4 DNA polymerase, and 6 µl (60 U) NEB T4 polynucleotide kinase. The polishing reaction was incubated in a thermal cycler for 20 min at 12°C, 20 min at 25°C, and than 20 min at 75°C. The samples were allowed to cool to room temperature while the ligation master mix was created. 4µl of 500 µM gSel3/4 adapter was added to each room-temperature genomic DNA sample. The conditions for adapter annealing and primer sequences were as published previously (Albert et al. 2007). The ligation master mix consisted of 8 µl 10 x NEB T4 polymerase buffer (NEB2), 4 µl 100 mM rATP, 51 µl water and 10 µl NEB T4 DNA ligase (400U/µl). The final ligation reaction volume was 200 µl (119 µl of polished DNA, 4 µl of both adapters, and 73 µl of ligation master mix). The reaction was incubated at room temperature for 90 min. Samples were prepared for small-fragment removal (e.g., adapter-dimer) by adding 2 µl of 10% (v/v) Tween-20 (Sigma, St. Louis, MO) and mixing. The samples were brought up to 300 µl total volume with TE (pH 8.0). Small DNA fragments were removed by adding 0.7 volume of AmpPure SPRI Beads (Agencourt Biosciences, Beverly, MA), vortexing, and incubating at room temperature for 5 min. The beads were collected using a rare earth magnetic particle collector (MPC, Invitrogen, Carlsbad, CA). The supernatant containing the small DNA fragments was removed by pipetting, and the bead pellets were washed twice with 500 µl of 70% (v/v) EtOH. The beads were then air-dried at 37°C in the MPC for 5 min. The libraries were eluted from the SPRI beads by vortexing in 50 µl of water. The SPRI beads were collected using the MPC and the amount of DNA was determined using a BioPhotometer (Eppendorf, Westbury, NY) The DNA quality control was performed on a BioAnalyzer 2100 DNA7500 chip. Amplification competency was assessed by LM-PCR with primers (gSel3) complementary to the ligated adapters; sequences and conditions were as previously published (Albert et al. 2007).

**Supplemental Experimental Procedure 2**

**Capture Array Handling**

Hybridization was performed using microarrays merged with X1 mixer on the NimbleGen Hybridization System for 3 days at 42°C following the manufacturer’s recommended conditions. Human Cot1 (Invitrogen/Life Technologies, Carlsbad, CA) was used at a mass ratio of 20:1 vs. the library (e.g., 100 µg human Cot1 to 5 µg capture library). Arrays were washed using recommended conditions for array CGH, the mixer seals were broken under 100 ml of NimbleGen 1xWash Buffer II at 42°C. There were different washing steps started with 2 washes in NimbleGen 1xStringent Wash Buffer at 47.5°C for two 5 minutes and went on to three further washing steps from 1xWash Buffer I to 1xWash Buffer III. Each wash step was done at room temperature with 10 inversions between each of the steps.

After the last wash step with 1x Wash Buffer III, captured molecules were eluted from the slides using a NimbleGen Elution Station according to the manufacturer’s recommended conditions. Eluted molecules were purified with MinElute Purification Kit (Qiagen, Venlo, NL) following the supplier´s instructions and amplified with the gSel adapters as previously described (Albert et al. 2007).

Quantitative PCR (SYBR-Green based; LC480 instrument) using four internal NimbleGen control loci (NSC-0237, NSC-0247, NSC-0268, NSC-0272) was performed to estimate relative fold-enrichment. Additionally, qPCR assays specific for three capture loci (b-raf exon15, k-ras c12 exon1, pten exon5) were used as positive controls to evaluate array capture efficiency.

**Supplemental Experimental Procedure 3:**

**Data Analysis - Variant Detection and Annotation.**

High Confidence Differences were calculated using the GS Reference Mapper assembly package (version 2.0.00.20; Roche Diagnostics) and hg18 reference sequence and SNPdb built 130.

The GS Reference Mapper application uses a combination of flow signal information, quality score information and difference type information to determine if a difference is High-Confidence. The general rules are:

• There must be at least 3 non-duplicate reads with the difference, unless the -e option is specified, in which case at least 10% of the expected depth must have the difference

• There must be both forward and reverse reads showing the difference, unless there are at least 5 reads with quality scores over 20 (or 30 if the difference involves a 5-mer or higher)

• If the difference is a single-base overcall or undercall, then the reads with the difference must form the consensus of the sequenced reads (i.e., at that location, the overallconsensus must differ from the reference) and the signal distribution of the differing reads must vary from the matching reads (and the number of bases in that homopolymer of the reference).

Trimmed, filtered and mapped bases were annotated as follows: (i) novelty based on overlap with the SNP database (build 130) (ii) effect on the encoded protein. The Ensembl hg18 (release May 2009) was used as reference sequence, SNP build 130 and gene coding information were collected from the University of California Santa Cruz genome browser ([http://genome.ucsc.edu](http://genome.ucsc.edu/)). Manual curation was done to (iii) identify and reject false positive sequence variants due to obvious sequence homology (>99% match to other genomic loci) of captured/cross-hybridised off-target sequences by BLAST analysis and to (iv) evaluate and reject putative in/del variants due to homopolymer sequencing artifacts and (v) select variants with ≥25% variation frequency (Var Freq).

**Supplemental Experimental Procedure 4:**

**Affymetrix SNP 6.0 Array Processing and Analysis**

Affymetrix GeneChip Human Mapping SNP 6.0 arrays were performed as described in the Genome-Wide Human SNP Nsp/Sty 6.0 User Guide (Affymetrix Inc., Santa Clara, CA, USA). In brief, 250 ng of genomic DNA was digested in parallel with Sty I and Nsp I restriction enzymes (New England Biolabs, Beverly, MA, USA). Adaptors Sty I or Nsp I were then ligated to the DNA ends. After dilution, samples were subjected to PCR. PCR was done by using Titanium Taq DNA polymerase (Clonetech); three PCR reactions for Sty I digested products and four reactions for Nsp I digested products. PCR product sizes were checked on a 2% agarose gel (1xTAE) by electrophoresis. Sty I and Nsp I products of each sample were combined, pooled and purified by using Agencourt Magnetic Beads (Beckman Coulter, Fullerton, CA, USA). Purified PCR products were quantitated and approximately 270 µg were fragmented and end-labeled with biotin. Labelled DNA was hybridized onto Genome-Wide Human SNP 6.0 Array at 50°C for 18h at 60 rotations per minute. The hybridized array was washed, stained, and scanned according to the manufacturer's (Affymetrix) instructions using Affymetrix GeneChip Command Console (AGCC, version 1.1).

SNP 6.0 data were imported and normalized using the Genotyping Console 4.0 program default settings. All samples passing QC criteria were subsequently genotyped using the Birdseed (v2) algorithm. We used 60 raw HapMap data generated with the Affymetrix Genome-Wide Human SNP Array 6.0 as reference. Data were obtained from Affymetrix (Affymetrix, Santa Clara, CA) web site and used for normalization.
